# Health-Promoting Phytobiotic-Based Feed Additive Improves Skin and Gill Proteome Response of Infected Fish

**DOI:** 10.3390/ani16091348

**Published:** 2026-04-28

**Authors:** Elissavet A. Arapi, Laura Fernández-Alacid, Maria Mercè Isern-Subich, Waldo G. Nuez-Ortín, Antoni Ibarz, Jo Cable

**Affiliations:** 1 School of Biosciences, Cardiff University, Cardiff CF10 3AX, UK; cablej@cardiff.ac.uk; 2 Department of Cell Biology, Physiology and Immunology, Faculty of Biology, University of Barcelona, Avda. Diagonal 643, 08028 Barcelona, Spain; fernandez_alacid@ub.edu (L.F.-A.); tibarz@ub.edu (A.I.); 3 Adisseo France S.A.S., Immeuble Antony Parc 2 10, Place du Général de Gaulle, 92160 Antony, France; mariamerce.isern@adisseo.com (M.M.I.-S.); waldo.nuezortin@adisseo.com (W.G.N.-O.)

**Keywords:** infectious diseases, feed additives, immunostimulants, phytobiotics, proteomics

## Abstract

Skin is a multifunctional organ essential for maintaining body homeostasis, regulating functions and providing protection from environmental stressors. In fish, skin is immune active, containing antimicrobial proteins acting as the first line of defence against infectious pathogens. The gills function similarly, as a key mucosal immunity site, where pathogens induce both innate and adaptive immune responses. In this study, proteomic analysis identified differentially expressed proteins in the skin and gills of ectoparasite *Gyrodactylus turnbulli* infected guppies (*Poecilia reticulata*) at two timepoints post-infection (Days 13 and 17). These fish were provided with a health-promoting additive, aimed to boost immunocompetency and reduce ectoparasite infections. Different proteomes were evidenced based on infection status of fish (susceptible, responding, or resistant) and in-feed supplementation. In skin tissue, susceptible fish showed no evidence of immune response, reflecting their high parasite load. Responding fish employed biological processes like apoptosis, reducing the gyrodactylid niche. In resistant fish, up-regulated innate and adaptive immunity explained the low parasite load on the host over the entire infection trajectory. Overall, fish protein expression in the skin and gills was affected both by the dietary supplement and gyrodactylid infection burden, highlighting the role of natural immunostimulants in aquatic infectious disease prophylaxis, control and treatment.

## Simple Summary

Plant-based compounds have gained attention in recent years as safer alternatives to conventional treatments for infectious diseases, which can pose risks to both hosts and the environment. In this study, we examined the effects of natural dietary supplementation on the skin and gills of infected guppies (*Poecilia reticulata*). Guppies were fed a diet enriched with organic acids, inactivated yeast and yeast extracts, herbal extracts and essential oils prior to controlled infection. This supplement, developed to mitigate parasite impacts on fish productivity and survival, has previously been shown to reduce *Gyrodactylus turnbulli* burden. We therefore hypothesised that the health-promoting feed additive would enhance tissue responses in infected fish. Using comparative proteomics, an approach increasingly applied in aquaculture to assess responses to stressors such as salinity, temperature, diet and infection, we identified immune-relevant proteins at two post-infection timepoints, revealing distinct proteomic profiles and a convergent immune response over time. Overall, we demonstrate that natural supplementation can both reduce parasite load and strengthen protective skin immune pathways, highlighting a practical dietary strategy for ectoparasite control in aquaculture.

## 1. Introduction

The skin is a complex, multi-tissue organ essential for homeostasis in all animals [1], acting as a barrier that maintains internal balance by regulating the exchange of ions, gases, nutrients, and metabolic substrates [1]. It also supports temperature regulation, protection from environmental challenges [2], and communication of species, gender, and social status [3], adapted to both terrestrial and aquatic environments. In fish, skin is a conserved, multifunctional organ that provides protection, communication, locomotion, respiration, excretion and sensory perception [4] by maintaining body shape, improving hydrodynamics and providing protection from shocks and attacks [5]. Even though fish skin is considered a relatively inert surface, it has an active role as a transport epithelium and a site of mucosal immunity [5], attributes also ascribed to the gills and gut [6]. Mucus, a complex medium, acts as a physical, dynamic, and semi-permeable barrier [7,8], which reduces the uptake of microbes and foreign substances [9,10,11], with antimicrobial properties demonstrated in many fish species [12,13,14,15,16]. Mucus is part of the innate immune response [10,17], acting as the first line of defence against pathogens, followed by the adaptive immune system, the ability to recognise and create immunological memory, characterised by slow cell proliferation as well as maturation and memory of lymphocytes [5,18].

The gills, another key site of mucosal immunity in fish, encounter pathogens capable of triggering both innate and adaptive immune responses [6,19]. Regulated immune responses are imperative to maintaining tissue homeostasis and integrity as adverse conditions are commonly characterised by inflammation and epithelial cell hyperplasia [19]. This was demonstrated in rainbow trout (*Oncorhynchus mykiss*; Walbaum, 1792) gills when infection by the parasitic ciliate *Ichthyophthirius multifiliis* (Fouquet, 1876) was characterised by intensive activity of immune cells (B-cells, T-cells and macrophages) and humoral elements (such as immunoglobulins) orchestrated by cytokines [20].

For this study, we used the model host–parasite system, the guppy (*Poecilia reticulata*; Peters, 1859), and its associated ectoparasite *Gyrodactylus turnbulli* (Harris, 1986). It has been extensively studied, including in regard to the Major Histocompatibility Complex (MHC) aspects of immunity [21,22]. In guppies, infection triggers an initial localised innate immune response at the parasite attachment site [23,24]. Inflammatory responses regulated by pro- and anti-inflammatory cytokines, such as TGF-β, are responsible for the proliferation of mucous and epithelial cells that are the main nutrient source for gyrodactylids [25,26]. Thus, nutrient depletion caused by down-regulation of cytokines could lead to parasite starvation which, coupled with host complement [27]—a second-line defence—could potentially lead to the elimination of parasites from a host. All hosts exposed to gyrodactylids tend to respond in one of three ways, namely resistant, responding, and susceptible [23], yet the mechanisms driving these different responses have never been investigated.

In recent years, plant-based compounds have gained traction as safer alternatives to traditional chemotherapeutics, which pose risks to both hosts and the environment [28,29]. Growth, mucosal immune responses and the total carotenoid content of juvenile guppies (*Poecilia reticulata*) fed 10 different herbal supplements have been shown to increase [30]. Studies on *Gyrodactylus turnbulli*-infected guppies have similarly shown that natural additives can boost immunity and reduce parasite load.

Comparative proteomic analysis is increasingly used in aquatic animals to assess responses to stressors such as salinity, temperature, diet and infection [31,32,33,34]. In aquaculture, it has helped identify immune-related proteins in fish exposed to pathogens, including *Ichthyophthirius multifiliis*-infected carp (*Cyprinus carpio*; Linnaeus, 1758) and *Edwardsiella ictalurid*-infected yellow catfish *(Pelteobagrus fulvidraco*; Richardson, 1846), where immune and signal transduction proteins were differentially regulated [11,35]. Dietary interventions also influence proteomic profiles: dietary carotenoids affected skin coloration of large yellow croaker (*Larimichthys crocea*; Richardson, 1846 [36]), phosphorus supplementation altered liver proteomes in infected obscure puffer (*Takifugu obscurus*; Abe, 1949; [37]), while functional additives enhanced immune responses in sea lice-infected Atlantic salmon (*Salmo salar*; Linnaeus, 1758; [38]). These findings suggest diet plays a significant role in modulating fish immunity at the proteomic level.

Despite growing evidence that plant-based supplements can reduce parasite burden, the underlying effects on host tissues remain poorly understood. Specifically, how such dietary interventions modulate proteomic responses during ectoparasitic infection has not yet been comprehensively explored. To address this gap, we investigated the impact of phytobiotic-based supplementation on the skin and gill proteome of *Gyrodactylus turnbulli*-infected guppies (*Poecilia reticulata*). While this supplement has previously been shown to reduce *G. turnbulli* burden [39], its mechanistic effects at the proteome level remain unclear. We therefore hypothesised that this health-promoting feed additive would modulate and enhance tissue-specific proteomic responses in infected fish.

## 2. Materials and Methods

### 2.1. Host and Parasite Origins and Maintenance

For this experiment, we used wild-type guppies originating from the Lower Aripo River, Trinidad, in 2010. Following transfer from the University of Exeter to Cardiff University, fish were maintained in the Cardiff Aquarium in 70 L tanks containing dechlorinated water, at an approximate density of 1 fish per L. For this experiment, 80 guppies were size-matched (standard length 17.2 ± 3.8 mm), divided into two treatments and maintained individually in 1 L containers, at 24 ± 1 °C, on a 12:12 h light: dark regime (07:00–19:00 h). Fish were fed daily (approx. 2% of their body weight) for two weeks with an Adisseo commercial feed (Adisseo; Antony, France), either with (n = 40) or without (control; n = 40) the Apex active ingredients. Apex is a phytobiotic-based functional feed additive that reduces the impact of fish ectoparasites [39]. Apex^®^ comprises a mixture of organic acids, inactivated yeast and yeast extracts, herbal extracts and essential oils on a mineral carrier, designed to mitigate the effects of monogenean gill parasites on fish productivity and survival by promoting fish immune competency (Adisseo, France). Due to confidentiality agreements, the proportions of ingredients included in the feed formulation cannot be disclosed. Then, each fish was briefly anaesthetised with MS-222 (0.02% tricaine methanesulfonate; Sigma-Aldrich; Dorset, UK) and infected with two *Gyrodactylus turnbulli* worms (strain Gt3) from an infected guppy donor (Day 0 of infection).

**Figure 1:**
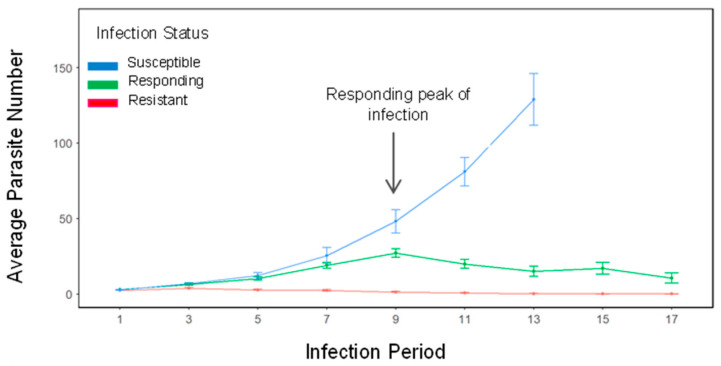
Average infection trajectories of guppies (*Poecilia reticulata*; n = 80) in each *Gyrodactylus turnbulli* infection group. Susceptible fish (blue) have an increasing parasite number throughout the infection period and would reach a fatal point. Responding fish (green) have a peak in parasite number around Days 9 to 13 (>10 parasites, Day 9 in this experiment), after which it declines. Resistant fish (red) have a low parasite load (<10 parasites) throughout the 17-day period. Error bars represent standard error.

The Gt3 gyrodactylid strain was established from ornamental fish originating from a UK pet store in 1997. Since then, a parasite culture was created and maintained under lab conditions. For experimental infections, a heavily infected donor fish was euthanised by overdose with MS 222, followed by pithing. The donor was then placed near an anaesthetised recipient fish, allowing direct contact and transfer of *G. turnbulli* from the caudal fin of the donor to that of the recipient. All infection procedures were observed under a dissecting microscope with fibre optic illumination, a process taking less than 30 s per fish. The recipient fish, then returned to dechlorinated water, took less than 10 s to recover. Fish were screened again the next day (Day 1) to confirm that the infection was successful and subsequently every 48 h for 17 days, during which the parasite number was recorded. Fish continued to be fed daily according to their treatment. After 17 days, fish from both treatments were divided into three categories based on their infection status: susceptible fish (blue; Figure 1), whose parasite load kept increasing and most likely reached fatal point; responding fish (green; Figure 1), whose parasite number peaked (>10 parasites) around Days 9 to 13 but then drastically dropped by Day 17; and resistant fish (red; Figure 1) with a low parasite load of less than 10 gyrodactylids over the infection period [23]. From the current study, we could not statistically analyse the proportion of fish which fell into the three categories with any reliability because proteomic sampling on Day 13 reduced the sample size; therefore, we analysed the data from [39], which used the same experimental protocol, and present the values from the current study.

### 2.2. Parasite Infection Statistical Analysis

Parasite metrics examined for statistical analyses were mean and maximum parasite number over the 17-day infection trajectory. Using the ‘lme4’ library [40], two Generalised Linear Models (GLMs) were constructed to assess which treatment had the best efficacy. For mean parasite number, the models were fitted with a ‘Gamma’ family and ‘log’ link function, whereas for maximum parasite number, the model was fitted with ‘quassipoisson’ family and ‘identity’ link function, and treatment was the fixed variable. As fish for both treatments were size-matched at the start of the feed trails, size variability was not considered further for analyses. For comparison of the three infection status groups (susceptible, responding, and resistant), a Kruskal–Wallis rank sum test was conducted, and Dunn’s post-hoc test indicated that there are no significant differences between any of the groups within treatment. For all models, error families were chosen based on the lowest AIC (Akaike Information Criterion) values, and the robustness of the models was assessed with visual examination of model plots to check standardised residuals for normal distribution and homogeneity of variance [40,41]. In all tests, the level of significance was taken as *p* < 0.05. All statistical analyses were performed in the R statistical software v4.1.1 [42].

### 2.3. Tissue Extraction

For tissue collection, skin and gill samples were collected on Day 13 (n = 50) and Day 17 (n = 30) post-infection. Fish were overdosed with MS-222, pithed, and rinsed in water, and then the skin was carefully extracted from both sides of the fish (from the operculum to the caudal fin) and gills from both sides were removed. Simultaneously, storage vials were pre-cooled in liquid nitrogen by snap freezing; the skin and samples were then placed into pre-cooled vials individually, which were sealed and dropped into liquid nitrogen before being transferred to a −80 °C freezer. All equipment was carefully cleaned with ethanol between samples. Frozen samples were transported on dry ice to the University of Barcelona, where protein extraction and quantification were conducted.

### 2.4. Protein Extraction

Skin and gill samples were homogenised in a lysis buffer (10 mL/g tissue; 7 M urea, 2 M thiourea, 2% *w*/*v* CHAPS and 1% protease inhibitor mixture) and centrifuged at 20,000 *g* for 15 min at 4 °C [9]. The supernatant was aliquoted, avoiding the surface lipid layer, while the pellet was resuspended. Subsequently, the protein concentration was determined using the Bradford assay [43], where bovine serum albumin (BSA; 1.42 mg/mL in a 2-fold serial dilution) was used to produce a standard curve, so concentration of samples could be determined at λ = 596 nm with a microplate reader (Infinity Pro200 spectrophotometer; Tecan, Spain). Cleaned and purified protein extracts were resuspended in the appropriate final volume of lysis buffer to achieve 1 μg/μL for each extract. Once the protein concentration of each sample was established (15.1–252.45 μg), guppy skin and gill tissue samples were pooled in groups of three individuals according to their infection status resulting in 4–5 pooled samples per infection group to ensure sufficient protein yield. Samples were then stored at −80 °C until further analysis.

### 2.5. Protein Identification and Quantitation

Protein identification and quantitation were performed by nano LC-MS/MS (Nanoscale liquid chromatography coupled to tandem mass spectrometry) at the University of Porto. The Ultimate 3000 liquid chromatography system coupled to a Q-Exactive Hybrid Quadrupole—Orbitrap mass spectrometer (Thermo Scientific; Bremen, Germany) was used as described in [44]. In brief, for each sample, 500 ng of protein was loaded onto a trapping cartridge (Acclaim PepMap C18 100 Å, 5 mm × 300 µm i.d., 160454, Thermo Scientific; Bremen, Germany) in a mobile phase of 2% acetonitrile (ACN), 0.1% formic acid (FA) at 10 µL/min. After 3 min of loading, the trap column was switched in-line to a 50 cm × 75 µm inner diameter EASYSpray column (ES803, PepMap RSLC, C18, 2 µm, Thermo Scientific; Bremen, Germany) at 250 nL/min. Separation was achieved by mixing A: 0.1% FA and B: 80% ACN, 0.1% FA with the following gradient: 5 min (2.5% B to 10% B), 120 min (10% B to 30% B), 20 min (30% B to 50% B), 5 min (50% B to 99% B), and 10 min (hold at 99% B). The column was then equilibrated with 2.5% B for 17 min. Data acquisition was facilitated by X calibur 4.0 and Tune 2.9 software (Thermo Scientific, Bremen, Germany). The mass spectrometer was operated in the data-dependent (dd) positive acquisition mode, alternating between a full scan (*m*/*z* 380–1580) and subsequent Higher-energy Collisional Dissociation (HCD MS/MS) of the 10 most intense peaks from a full scan (normalised collision energy of 27%). The electrospray ionisation (ESI) spray voltage and the global, full scan and MS settings were as described in [44]

### 2.6. Interactome Analysis

The data obtained from nano LC-MS/MS analysis were assessed, and differentially expressed proteins were identified. For the selection of 2-fold differentially expressed proteins, three criteria were considered: protein abundance ratio in fish of the same infection status between treatments (Apex or control), abundance ratio *p*-value (*p* < 0.05) and protein peaks found in three or more out of the five samples. Protein identification analysis was performed with the data available in the UniProt protein sequence database v2022_03 [45]. Gene symbols and NCBI Entrez gene numbers were retrieved from Gene Cards v3 [46] using the Homo sapiens database and were then submitted to STRING Program v11.5 [47] to create protein interactomes. The mechanisms of response involving the DEPs were obtained from a comparative analysis using *Homo sapiens* (Linnaeus, 1758) as a reference organism in order to extract the maximum information currently available. Thus, an ortholog *H. sapiens* Entrez Gene ID was assigned based on sequence homology. Interactomes were derived from confidence analysis of the protein–protein interaction (PPI) networks with a confidence score of 0.300–0.500 and a PPI enrichment of *p* < 0.05. Such a PPI enrichment *p*-value indicates that proteins have more interactions among themselves than would be expected for a random set of proteins of the same size and degree distribution drawn from the genome, so proteins are at least partially biologically connected as a group. Protein–protein interaction networks were generated, where edges (lines) between proteins represented functional associations; thicker edges indicated higher confidence scores, and colours denoted different evidence sources. For this study, all differentially expressed proteins were identified and clustered according to their biological processes (Gene Ontology annotation (GO); [48]) and associated reactome pathways (Homo sapiens annotation (HAS); [49]), and the false discovery rate (fdr; controls the expected number of false positives among the positive test results) was recorded. As all guppies were infected and subdivided according to their infection status (susceptible, responding and resistant), the analysis focused on immune-relevant proteins in response to infection. The BioMart Ensembl v115 tool [50] confirmed that 86.6% of the unique Gene IDs identified in this study have orthologue genes in zebrafish (*Danio rerio*; Hamilton, 1822), verifying the use of *Homo sapiens* as a reference organism.

## 3. Results

**Figure 2:**
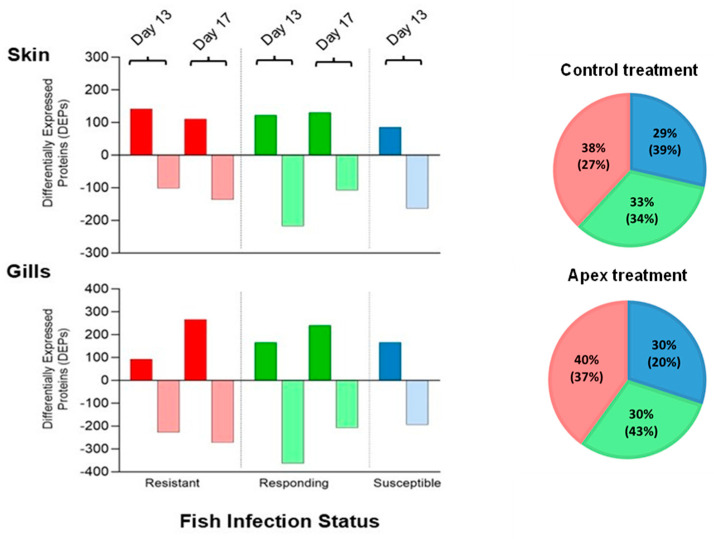
Distribution of differentially expressed proteins (DEPs) among resistant, responding and susceptible guppies (*Poecilia reticulata*) infected with *Gyrodactylus turnbulli* when supplemented with the Apex supplement over the control diet. DEPs in both skin and gill tissue on Day 13 and Day 17 post-infection with up-regulated proteins depicted above the zero line and down-regulated proteins below the zero line. Pie charts illustrate the relative proportions of susceptible (blue), responding (green) and resistant (red) fish on Day 13 of infection. From the current study, sample sizes were too small to support statistical analysis. Therefore, we analysed proportions of infection status groups from [39], in which no significant difference was found between the Apex and control diets (χ^2^ = 0.98, *p* = 0.61). Values from that previous study are shown in parentheses within each pie chart.

Guppies supplemented with Apex had a significantly lower maximum parasite number during the infection period by 20% (GLM; *p* < 0.001), indicating the effectiveness of the in-feed treatment, consistent with the results reported in [39]. Across the three infection status categories, proportions did not differ between diets in the current study (Figure 2), although sample sizes in this study were too small for statistical analysis. We therefore analysed infection status proportions from [39], where no significant difference was detected between the Apex and control diets (χ^2^ = 0.98, *p* = 0.61). In that study, Apex-fed fish comprised 37% resistant, 43% responding and 20% susceptible individuals, whereas control-fed fish comprised 27% resistant, 34% responding and 39% susceptible individuals (Figure 2).

Overall, 3521 proteins were differentially expressed (DEPs) in the skin and gills of guppies fed the supplement compared to the control diet (Table 1). All samples collected for this study were derived from infected fish on two different diets, so there is no direct comparison with uninfected fish.

### 3.1. Skin and Gill Proteome Analysis

In skin tissue on Day 13 post-infection, 835 DEPs were identified in the supplemented fish over the control fish; 351 proteins were up-regulated and 484 were down-regulated (Figure S1). On Day 17 post-infection, out of the 487 DEPs identified, 242 proteins were up-regulated and 245 proteins were down-regulated in fish with the supplemented diet over the control (Figure S1).

In gill tissues on Day 13 post-infection, 1212 DEPs were identified in the supplemented fish over the control fish; 427 proteins were up-regulated and 785 were down-regulated (Figure S2). On Day 17 post-infection, out of the 987 DEPs identified, 507 proteins were up-regulated, and 480 proteins were down-regulated in fish with the supplemented diet over the control (Figure S2).

### 3.2. Infection Status (Susceptible, Responding and Resistant Fish)

We looked into the groups with different infection status, and the distribution of DEPs for susceptible, responding and resistant fish supplemented with Apex over the control diet at Days 13 and 17 is represented in Figure 2. A summary of all cluster reactomes related to immunity are presented in Table 1.

#### 3.2.1. Susceptible Fish: High Parasite Burden with Limited Immune Response

Susceptible fish had a high parasite load (average parasite burden: 152.6) that would most likely keep increasing and potentially become fatal to the host if the infection had not been terminated prematurely at Day 13; thus, no samples of susceptible fish were collected on Day 17.

When looking at DEPs in susceptible fish skin on Day 13, 86 proteins were up-regulated (avg. local clustering coefficient: 0.337; PPI enrichment *p* = 0.032; Figure S3) and 164 proteins were down-regulated (avg. local clustering coefficient: 0.358; PPI enrichment *p* = 2.09 × 10^−6^; Figure S1). In the up-regulated proteins, no biological processes or reactome pathways directly related to immunity were identified, so no significant clusters were built (Figure S3). In the down-regulated proteins, 79 were associated with disease (DOID:4; diseases gene association; fdr = 0.0011), without any specific biological processes or reactome pathways linked to immunity being activated.

**Figure 3:**
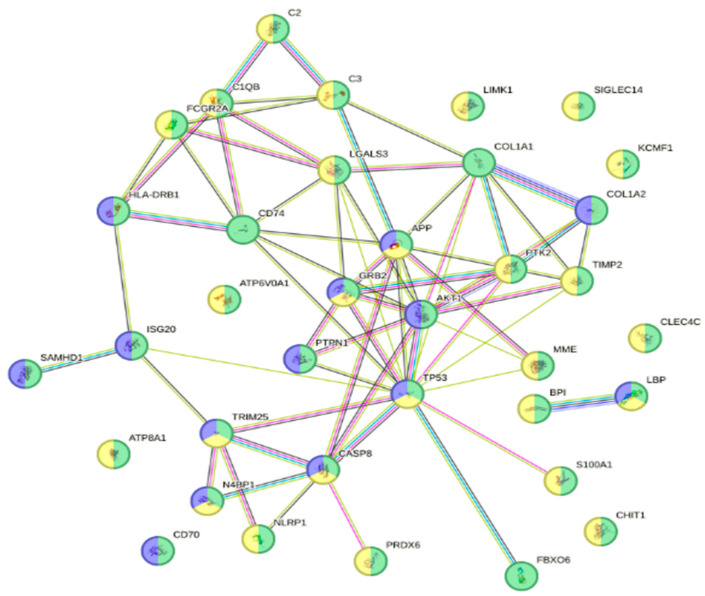
Interactome of the reactome pathway of the immune system with 36 up-regulated proteins (Table S1) in susceptible fish gills on Day 13 post-infection (green; HSA-168256; fdr = 5.88 × 10^−5^). Proteins were also involved in other reactome pathways such as innate immune system (yellow; HSA-168249; fdr = 4.45 × 10^−5^) and neutrophil degranulation (blue; HSA-6798695; fdr = 0.0144).

When looking at DEPs in susceptible fish gills on Day 13, 167 proteins were up-regulated (avg. local clustering coefficient: 0.412; PPI enrichment *p* = 2.16 × 10^−6^; Figure S4) and 194 were down-regulated (avg. local clustering coefficient: 0.362; PPI enrichment *p* = 9.8 × 10^−11^; Figure S4). In the up-regulated proteins, 36 proteins (Table S1) were associated with the reactome pathway of the immune system (green; HSA-168256; fdr = 5.88 × 10^−5^) and with other reactomes, such as the innate immune system (yellow; HSA-168249; fdr = 4.45 × 10^−5^) and neutrophil degranulation (blue; HSA-6798695; fdr = 0.0144), as indicated in Figure 3.

**Figure 4:**
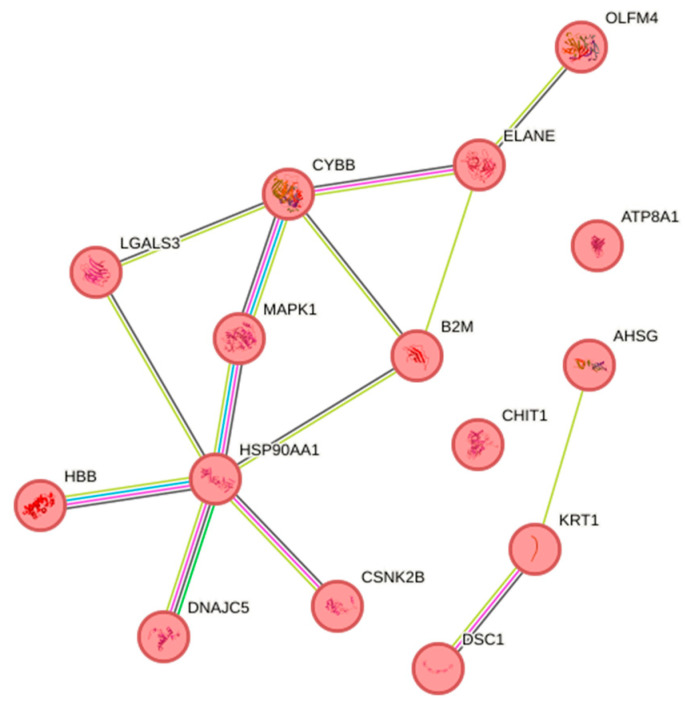
Interactome of the 15 down-regulated proteins (Table S2) in susceptible fish gills on Day 13 post-infection associated with the reactome pathway of neutrophil degranulation (red; HSA-6798695; fdr = 1.63 × 10^−21^).

In the down-regulated proteins, 81 proteins were associated with disease (DOID:4; diseases gene association; fdr = 0.0005). Of these, 15 (Table S2) were associated with the reactome pathway of neutrophil degranulation (red; HSA-6798695; fdr = 1.63 × 10^−21^) as shown in Figure 4. Despite this group having the highest parasite load, DEPs associated with response to stimulus, host immunity, cell death or other functions relevant to parasitism were only expressed in the gill and not skin tissue, where most of the parasites were located.

#### 3.2.2. Responding Fish: Shifting Proteomic Signatures Through the Infection Period

**Figure 5:**
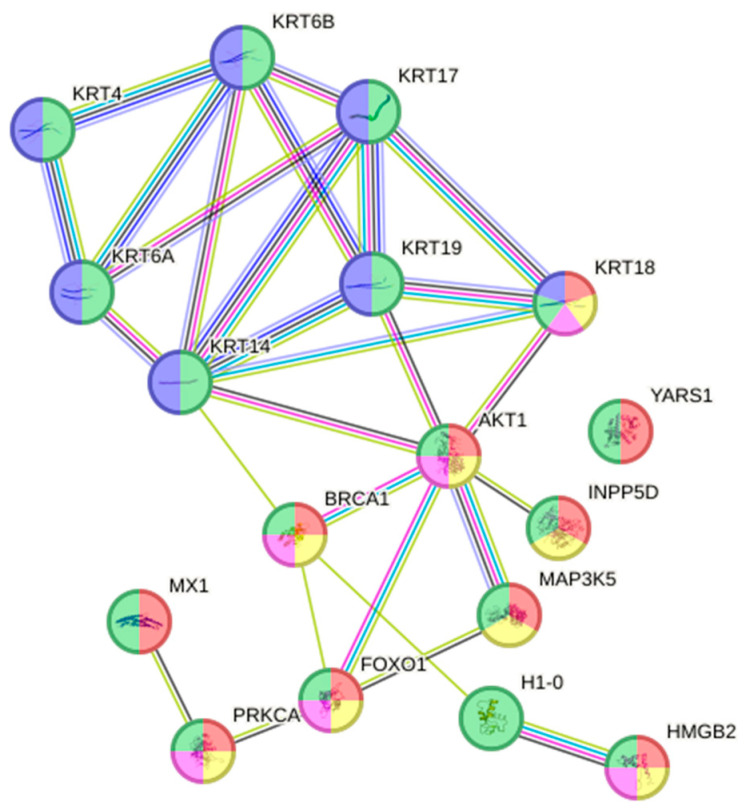
Interactome of the programmed cell death biological process with 17 up-regulated proteins (Table S3) in responding fish skin on Day 13 post-infection (green; GO:0012501; fdr = 0.020). Proteins were also involved in other biological processes like the apoptotic process (red; GO:0006915; fdr = 0.003), regulation of the apoptotic process (yellow; GO:0042981; fdr = 0.016), cornification (blue; GO:0070268; fdr = 0.0009) and negative regulation of the apoptotic process (purple; GO:0043066; fdr = 0.040).

**Figure 6:**
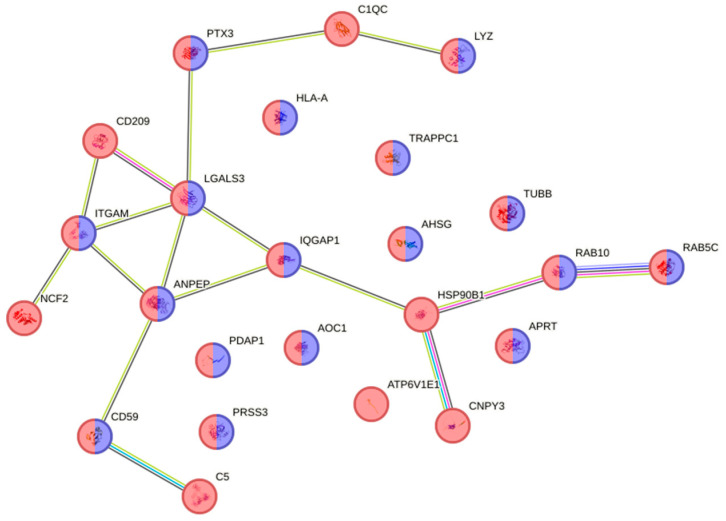
Interactome of the 24 down-regulated proteins (Table S4) in responding fish skin on Day 13 post-infection. Proteins were associated with the reactome pathways of innate immune system (red; HSA-168249; fdr < 0.0001) and neutrophil degranulation (blue; HSA-6798695; fdr = 0.001).

The parasite load of responding fish (>10 parasites) peaked around Days 9 to 13, but then dropped, indicating an active immune response function. When looking into fish skin tissue on Day 13, 123 proteins (avg. local clustering coefficient: 0.494; PPI enrichment *p* = 0.0003) were up-regulated (Figure S3). Of these, 17 proteins (Table S3) were grouped according to their biological process of programmed cell death (green; GO:0012501; fdr = 0.020; Figure 5). Interestingly, some of the proteins involved in the programmed cell death process were also associated with other similar biological processes, such as the apoptotic process (red; GO:0006915; fdr = 0.003), the regulation of the apoptotic process (yellow; GO:0042981; fdr = 0.016), cornification (blue; GO:0070268; fdr = 0.0009) and negative regulation of the apoptotic process (purple; GO:0043066; fdr = 0.040), as shown in Figure 5. When looking at proteins down-regulated in responding fish skin tissue, 24 (Table S4) out of 218 proteins (avg. local clustering coefficient: 0.473; PPI enrichment *p* < 0.0001; Figure S3) were clustered in the reactome pathway of the innate immune system (red; HSA-168249; fdr < 0.0001; Figure 6). Some proteins of the innate immune system pathway were also involved in the reactome pathway of neutrophil degranulation (blue; HSA-6798695; fdr = 0.001) as seen in Figure 6. 

In gill tissue on Day 13, 363 proteins (avg. local clustering coefficient: 0.375; PPI enrichment *p* < 1.0 × 10^−16^; Figure S4) were down-regulated and 167 (avg. local clustering coefficient: 0.331; PPI enrichment *p* = 0.0245; Figure S4) were up-regulated. In the up-regulated proteins, no biological processes or reactome pathways directly related to immunity were identified. In the down-regulated proteins, 131 were associated with disease (DOID:4; disease-gene association; fdr = 0.0166) but no biological processes or reactome pathways were directly linked to immunity.

**Figure 7:**
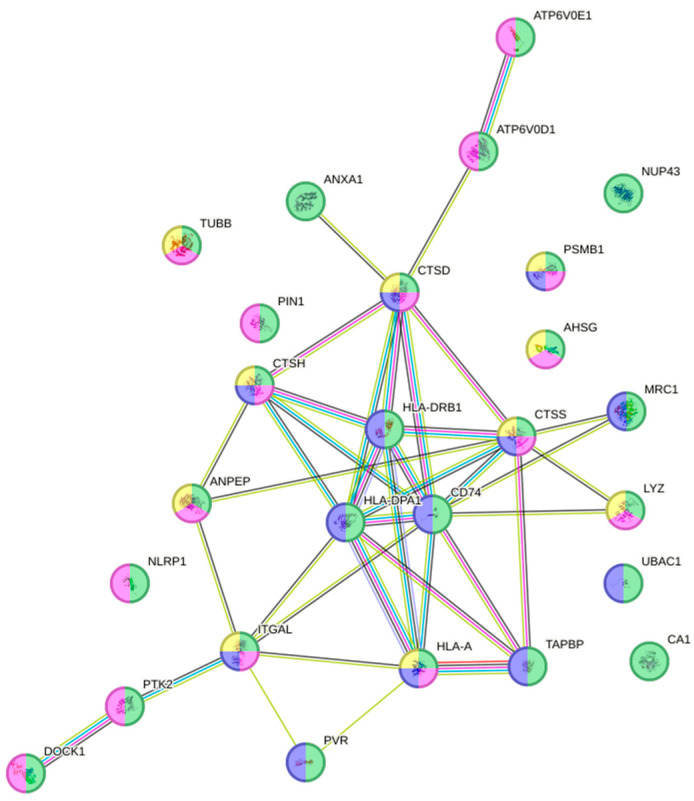
Interactome of the reactome pathway of the immune system with 26 up-regulated proteins (Table S5) in responding fish skin on Day 17 post-infection (green; HAS-168256; fdr = 3.02 × 10^−23^). Proteins were also involved in the reactome pathways of the innate immune system (purple; HSA-168249; fdr = 1.51 × 10^−11^), adaptive immune system (blue; HSA-1280218; fdr = 2.23 × 10^−9^) and neutrophil degranulation (yellow; HSA-6798695; fdr = 1.60 × 10^−7^).

On Day 17, out of 131 up-regulated proteins in skin tissue (avg. local clustering coefficient: 0.388; PPI enrichment *p* = 1.72 × 10^−8^; Figure S5), 26 proteins (Table S5) were clustered in the reactome pathway of the immune system (green; HAS-168256; fdr = 3.02 × 10^−23^) and associated with other reactome pathways such as the innate immune system (purple; HSA-168249; fdr = 1.51 × 10^−11^), adaptive immune system (blue; HSA-1280218; fdr = 2.23 × 10^−9^) and neutrophil degranulation (yellow; HSA-6798695; fdr = 1.60 × 10^−7^), as seen in Figure 7. When looking into the 108 down-regulated proteins (avg. local clustering coefficient: 0.401; PPI enrichment *p* < 0.0005; Figure S5), there were no biological processes or reactome pathways directly linked to immunity. In gill tissues on Day 17, 241 proteins were up-regulated (avg. local clustering coefficient: 0.402; PPI enrichment *p* = 0.066; Figure S6) and 208 were down-regulated (avg. local clustering coefficient: 0.38; PPI enrichment *p* = 3.34 × 10^−6^; Figure S6). In both differentially expressed protein groups, no expression of biological processes or reactome pathways was directly linked to immunity.

#### 3.2.3. Resistant Fish: Effective Resistance by Sustained Immune Response Regulation

**Figure 8:**
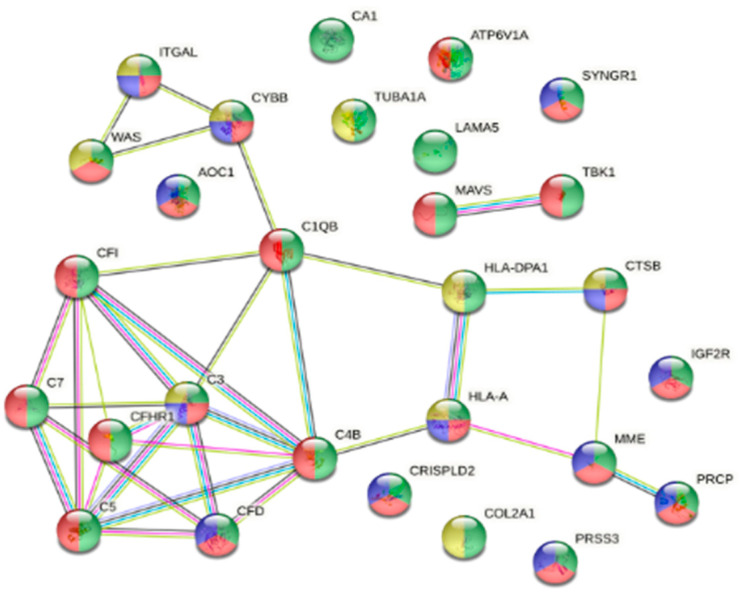
Interactome of the reactome pathway of the immune system with 28 up-regulated proteins (Table S6) in resistant fish skin on Day 13 post-infection (green; HSA-168256; fdr = 0.029). Proteins were also associated in reactome pathways of the innate immune system (red; HSA-168249; fdr = 0.0003), neutrophil degranulation (blue; HSA-6798695; fdr < 0.0001) and adaptive immune system (yellow; HSA-1280218; fdr = 0.0013).

Resistant fish had a low parasite load (<10 parasites) throughout the 13-day infection trajectory. On Day 13 in the skin tissue, from the 142 up-regulated proteins (avg. local clustering coefficient: 0.41; PPI enrichment *p* < 0.0001; Figure S3), 28 (Table S6) were associated with the reactome pathway of immune response (green; HSA-168256; *p* = 0.029; Figure 8). Some of the proteins clustered in the immune response were also clustered in other similar pathways such as the innate immune system (red; HSA-168249; fdr = 0.0003), neutrophil degranulation (blue; HSA-6798695; fdr < 0.0001) and adaptive immune system (yellow; HSA-1280218; fdr = 0.0013) as seen in Figure 8.

**Figure 9:**
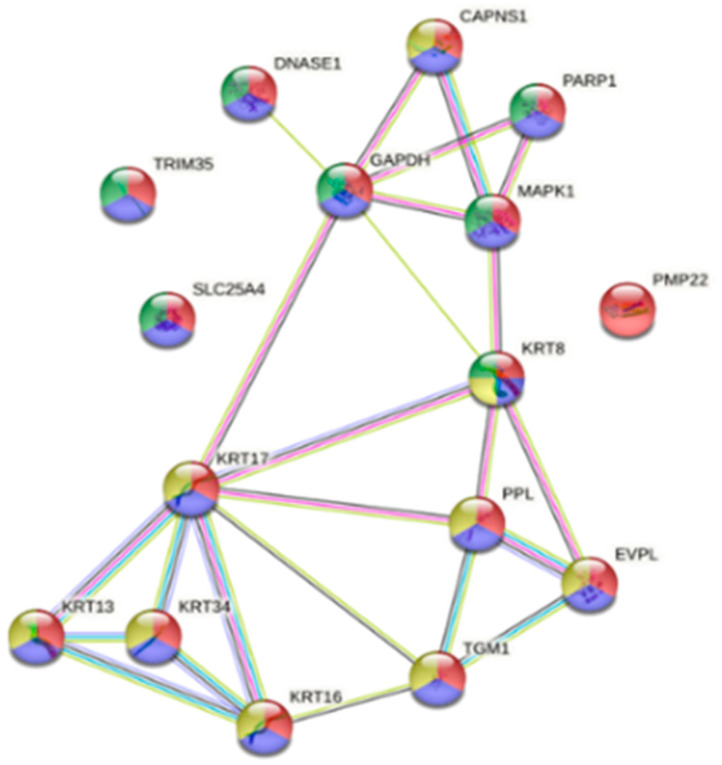
Interactome of the 16 down-regulated proteins (Table S7) in resistant fish skin on Day 13 post-infection associated with the biological process of cell death (red; GO:0008219; fdr = 0.0277), programmed cell death (blue; GO:0012501; fdr < 0.0001), apoptotic process (green; GO:0006915, fdr = 0.0044) and cornification (yellow; GO:0070268; fdr < 0.0001).

**Figure 10:**
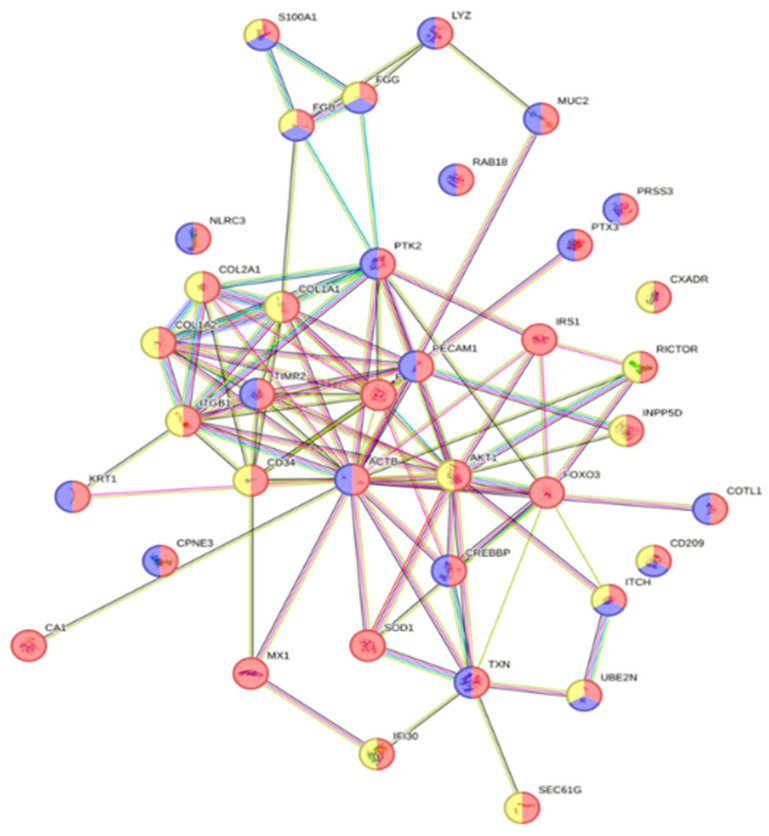
Interactome of the reactome pathway of the immune system with 38 down-regulated proteins (Table S8) in resistant fish gills on Day 13 post-infection (red; HSA-168256; fdr = 0.0027). Proteins were also linked to other reactome pathways such as the innate immune system (blue; HSA-168249; fdr = 0.0444) and adaptive immune system (yellow; HSA-1280218; fdr = 0.0425).

Of the 102 down-regulated proteins (avg. local clustering coefficient: 0.325; PPI enrichment *p* = 0.0002; Figure S3) in resistant fish skin, 16 (Table S7) were grouped in the biological process of cell death (red; GO:0008219; fdr = 0.0277; Figure 9). Some of the proteins were also linked with other biological processes such as programmed cell death (blue; GO:0012501; fdr < 0.0001), the apoptotic process (green; GO:0006915, fdr = 0.0044) and cornification (yellow; GO:0070268; fdr < 0.0001) as shown in Figure 9. In gill tissue of resistant fish on Day 13, 93 proteins were up-regulated (avg. local clustering coefficient: 0.355; PPI enrichment *p* = 0.00314; Figure S4), with no biological processes or reactome pathways directly linked to immunity. A total of 38 (Table S8) out of the 228 down-regulated proteins (avg. local clustering coefficient: 0.506; PPI enrichment *p* < 1.0 × 10^−16^; Figure S4) were linked to the reactome pathway of the immune system (red; HSA-168256; fdr = 0.0027; Figure 10). The same proteins were also linked to other reactome pathways, such as the innate immune system (blue; HSA-168249; fdr = 0.0444) and adaptive immune system (yellow; HSA-1280218; fdr = 0.0425), as shown in Figure 10.

**Figure 11:**
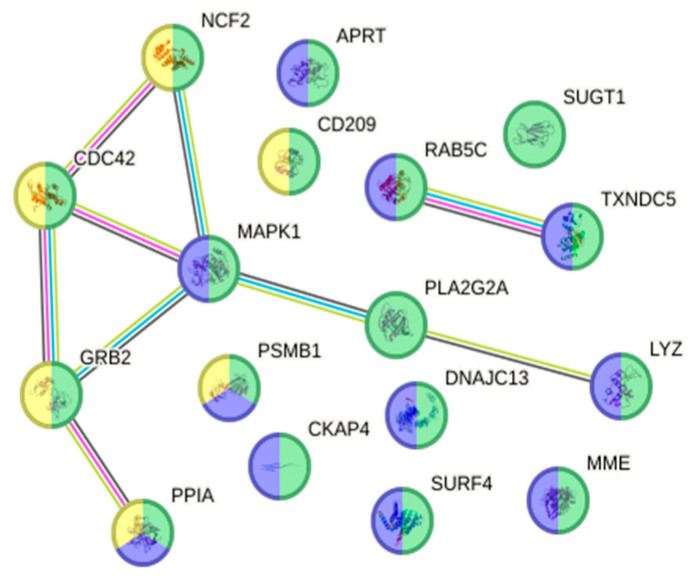
Interactome of the 17 up-regulated proteins (Table S9) in resistant fish skin on Day 17 post-infection associated with the reactome pathways of the innate immune system (green; HSA-168249; fdr = 5.13 × 10^−13^), neutrophil degranulation (blue; HSA-6798695; fdr = 1.54 × 10^−11^) and adaptive immune system (yellow; HSA-1280218; fdr = 0.0086).

On Day 17 in skin tissue, 111 proteins were up-regulated (avg. local clustering coefficient: 0.373; PPI enrichment *p* = 0.00228; Figure S5) and 137 proteins were down-regulated (avg. local clustering coefficient: 0.356; PPI enrichment *p* = 0.00314). In the group of up-regulated proteins, 17 (Table S9) were associated with the reactome pathway of the innate immune system (green; HSA-168249; fdr = 5.13 × 10^−19^), with the same proteins also linked other reactome pathways such as neutrophil degranulation (blue; HSA-6798695; fdr = 1.54 × 10^−11^) and the adaptive immune system (yellow; HSA-1280218; fdr = 0.0086), as shown in Figure 11. When looking into the down-regulated proteins, there was a significant correlation between DEPs and host immunity. In gill tissue of resistant fish on Day 17, 267 proteins were up-regulated (avg. local clustering coefficient: 0.386; PPI enrichment *p* = 2.22 × 10^−16^; Figure S6) and 272 were down-regulated (avg. local clustering coefficient: 0.384; PPI enrichment *p* = 6.01 × 10^−7^; Figure S6), with no biological processes or reactome pathways directly linked to immunity.

## 4. Discussion

This is the first study to explore the skin and gill proteome of guppies responding to *Gyrodactylus turnbulli* infection while comparing an Apex phytobiotic-based diet (Adisseo, France) with a control diet at Day 13 and Day 17 post-infection. In response to infection, fish were grouped into three different groups according to severity (susceptible, responding, and resistant according to [23]). Proteomic analysis revealed that the supplemented diet enhanced host immunity, reduced maximum parasite load by 20% and identified distinct differentially expressed proteins (DEPs) among susceptible, responding, and resistant fish, highlighting, for the first time, the mechanisms underlying their varied responses to infection.

In susceptible fish (average parasite burden: 152.6), no DEPs associated with immunity were identified, explaining why increasing parasite numbers lead to host mortality. Responding fish (with an infection load > 10) showed up-regulation of apoptosis and cornification, potentially leading to limitations regarding the density of epithelial and mucosal cells on which the parasites feed, leading to parasite elimination. Resistant fish (with an infection load < 10) exhibited an active immune response at both Days 13 and 17, resulting in a low gyrodactylid load throughout the infection period. As shown in [39], fish fed with Apex exhibited a 40% reduction in parasite load, with the supplemented diet effectively lowering peak parasite numbers in both susceptible and responding individuals. The skin of susceptible fish showed no evidence of DEPs associated with an immune response or response to stress on Day 13. At that point, there was already a high parasite load on the host, which would have typically led to host death by Day 17, as seen in [51], so for ethical reasons, all other fish in this category were euthanised early and sampling at Day 17 was not possible.

In responding fish skin, biological processes such as programmed cell death (GO:0012501), apoptosis (GO:0006915), regulation of the apoptotic process (GO:0042981) and cornification (GO:0070268) were up-regulated on Day 13. Such processes are essential physiological processes that play a critical role in controlling the development and removal of cells at the appropriate time throughout an organism’s life [52]. Apoptosis is a non-lytic and typically immunologically silent form of cell death, triggered in cases of damaged DNA and abnormal cells, whereas programmed lytic cell death is highly inflammatory, involves multiple stages and is activated by stressors, like disease and toxic agents [53,54]. Cornification is also a form of terminal differentiation that occurs in the epidermal keratinocytes [55,56]. These processes, involved in cell death, constitute a means of host defence employed by responding fish [57]. They benefit the host by limiting the accumulation of harmful or potentially dangerous cells and by eliminating the niche of certain pathogens [58]. In the case of gyrodactylids, such processes limit the density of epithelial and mucosal cells on which the parasites feed [59]. Therefore, one efficient strategy of responding fish regarding infection is host cell death, which ultimately leads to a reduction in the fish parasite load.

In resistant fish skin, innate (HSA-168249) and adaptive immunity (HSA-1280218) pathways, along with neutrophil degranulation (HSA-6798695), were up-regulated. Parasite-induced tissue damage exposes the skin, gills, and mucus to infection, where immune-relevant proteins, such as lysozyme, immunoglobulins, lectins, complement, and C-reactive proteins, and other antimicrobial enzymes play a key protective role [7,9]. These defence proteins, amongst other antimicrobial enzymes, have also been reported in infected fish, including Atlantic salmon (*Salmo salar*) challenged by sea lice (*Lepeophtheirus salmonis*; Krøyer, 1837; see [60]) and common carp (*Cyprinus carpio*) infected with white spot (*Ichthyophthirius multifiliis*; see [11]). More specifically, neutrophils, part of neutrophil degranulation, migrate to inflamed tissues in response to chemotactic signals and rapidly release granules to combat infection [61]. Similarly, this process has been observed in the skin mucus of yellow catfish (*Pelteobagrus fulvidraco*) infected with *Edwardsiella ictaluri* (Hawke et al., 1981), contributing to host defence [35]. By Day 17, resistant fish continued to show up-regulated immune pathways, while responding fish exhibited a complete shift in their proteomic profile compared to Day 13. At this stage, parasite loads in responding fish resembled those of resistant fish, resulting in a proteomic profile resembling that of the resistant group.

Overall, in gill tissue, immune-related pathways were upregulated only in susceptible fish on Day 13. With the increasing parasite number on the host, parasites started migrating from the tail towards the body and head, eliciting an up-regulation of the immune response on the gills that was only detected on Day 13. The apparent activation and inhibition of neutrophil degranulation exhibited in susceptible fish represent different regulatory stages of the immune response rather than simultaneous opposing processes. In contrast, in responding and resistant fish, there was minimal rostral movement of parasites on the host due to the lower parasite burden. As a result, there was no strong activation of immunity, potentially explaining the decrease in immune system regulation observed—a pattern that persisted through Day 17.

Enhancing skin and mucosal immunity through dietary supplementation has previously been shown to improve disease resistance in fish at the proteomic level [62]. This was also supported by the authors of [63], who demonstrated that dietary inclusion of spray-dried porcine plasma (SDPP) improved somatic growth, enhanced feed efficiency parameters, and supported the immune system of gilthead sea bream (*Sparus aurata*; Linnaeus, 1758) skin mucosa. As demonstrated in [64], even though immunity proteins were identified in greater amberjack (*Seriola dumerili*; Risso, 1810) mucus infected with the *Neobenedenia girellae* (Yamaguti, 1963) ectoparasite, infection progressed regardless of immunity. Therefore, the need for functional feed additives stimulating the immune system was highlighted [64].

Natural supplements like peppermint (*Mentha piperita*; Linnaeus, 1753) and neem (*Azadirachta indica*; de Jussieu, 1830) have demonstrated immunostimulant and antimicrobial benefits, improving survival in *Vibrio harveyi*-infected Asian seabass (*Lates calcarifer*; Bloch, 1790; [65,66]), and similar effects were observed when kernel (*Magnifera indica*; Linnaeus, 1753) was provided to Rohu fingerlings (*Labeo rohita*; Hamilton, 1822) infected with *Aeromonas hydrophila* (see [67]) and other species including Nile tilapia (*Oreochromis niloticus*; Linnaeus, 1758; see [68]), rainbow trout (*Oncorhynchus mykiss*; Linnaeus, 1758; see [69]) and European sea bass (*Dicentrarchus labrax*; Linnaeus, 1758; see [70]). In addition to stimulating the fish immune system and enhancing growth performance, health condition and disease prevention, natural compounds are considered more desirable for consumer consumption [71,72].

A limitation of this study is the absence of an uninfected control group, as all experimental fish were infected. Consequently, proteomic analyses compared infected fish fed a control diet, with infected fish receiving dietary supplementation. This design enabled evaluation of the effects of dietary supplementation under infection conditions but did not allow assessment of baseline proteomic responses to infection itself.

Given the promising effects of the Apex phytobiotic supplement, together with the antimicrobial benefits reported for other natural compounds, future research should focus on plant-based formulations to determine whether observed benefits arise from specific bioactive constituents, synergistic interactions among ingredients, or formulation-dependent properties, thereby informing optimisation of dietary interventions. In addition, evaluating long-term supplementation is essential to establish whether these effects are sustained or attenuate over time, and to assess potential physiological or energetic trade-offs associated with prolonged immune stimulation. Such work would clarify whether enhanced parasite resistance confers durable improvements in health, growth efficiency, and overall resilience under extended production conditions.

## 5. Conclusions

This study is the first to analyse the skin and gill proteome of *Gyrodactylus turnbulli*-infected guppies. Immune-relevant proteins were identified in fish fed Apex at Days 13 and 17 post-infection, revealing distinct proteomic profiles among susceptible, responding, and resistant individuals. While coping mechanisms varied at Day 13, a convergent immune response emerged by Day 17. Natural, phytobiotic supplementation can both lower gyrodactylid peak loads and boost protective skin immune pathways, highlighting a feasible dietary strategy for ectoparasite control in aquaculture.

## Figures and Tables

**Table 1:** Summary of results. Differentially expressed proteins (DEPs) in skin and gill tissues of *Gyrodactylus*-infected guppies *(Poecilia reticulata)* provided with the phytobiotic-based Apex additive versus the control diet on Day 13 (peak of the infection trajectory) and Day 17 (last day of the infection trajectory). At this second sample point, susceptible fish had reached a fatal point and were not available for sampling, whereas resistant and responding fish were clearing their infection. All cluster reactomes related to immunity are summarised in this table.

		DEPs Day 13	Cluster Reactomes	DEPs Day 17	Cluster Reactomes
Susceptible fish	**Skin Tissue**	86 up-regulated	*No immune clusters*	-	-
		164 down-regulated	Disease	-	-
	**Gill Tissue**	167 up-regulated	Immune system Innate immune system Neutrophil degranulation (Figure 3) (Table S1)	-	-
		194 down-regulated	Disease Neutrophil degranulation (Figure 4) (Table S2)	-	-
Responding fish	**Skin Tissue**	123 up-regulated	Cell death and apoptotic process Regulation of apoptotic process Negative regulation of apoptosis Cornification (Figure 5) (Tables S3)	131 up-regulated	Immune System Innate immune system Adaptive immune system Neutrophil degranulation (Figure 7) (Table S5)
		218 down-regulated	Innate immune system Neutrophil degranulation (Figure 6) (Table S4)	108 down-regulated	*No immune clusters*
	**Gill Tissue**	363 up-regulated	*No immune clusters*	241 up-regulated	*No immune clusters*
		167 down-regulated	Disease	208 down-regulated	*No immune clusters*
Resistant fish	**Skin Tissue**	142 up-regulated	Immune system Innate immune system Adaptive immune system Neutrophil degranulation (Figure 8) (Table S6)	111 up-regulated	Innate immune system Adaptative immune system Neutrophil degranulation (Figure 11) (Table S9)
		102 down-regulated	Cell death Apoptotic process Programmed cell death Cornification (Figure 9) (Table S7)	137 down-regulated	*No immune clusters*
	**Gill Tissue**	93 up-regulated	*No immune clusters*	267 up-regulated	*No immune clusters*
		228 down-regulated	Immune system Innate immune system Adaptative immune system (Figure 10) (Table S8)	272 down-regulated	*No immune clusters*
**Total** **DEPs**	**Skin Tissue**	351 up-regulated	(Figure S1)	242 up-regulated	(Figure S1)
		484 down-regulated	(Figure S1)	245 down-regulated	(Figure S1)
**Total** **DEPs**	**Gill Tissue**	427 up-regulated	(Figure S2)	507 up-regulated	(Figure S2)
		785 Down-regulated	(Figure S2)	480 down-regulated	(Figure S2)

## Data Availability

Data is available in a publicly accessible repository: https://data.mendeley.com/preview/9thhgn4g94?a=61444942-1b3e-496e-a9c4-a797af927c1e (accessed on 4 March 2026).

## Supplementary Materials

The following supporting information can be downloaded at https://www.mdpi.com/article/10.3390/ani16091348/s1, Tables S1–S9: Lists of the differentially expressed proteins associated with *Gyrodactylus turnbulli* infected guppies; Figures S1 and S2: Interactomes of proteins differentially expressed in skin and gill tissue of *Gyrodactylus* infected guppies; Figures S3–S6: Interactomes of proteins differentially expressed in skin and gill tissue of the guppy infection status groups at Days 13 and 17 post-infection.
