# Supplementary material for: Health-Promoting Phytobiotic-Based Feed Additive Improves Skin and Gill Proteome Response of Infected Fish
Source: Animals (Basel). 2026 Apr 28;16(9):1348. doi: 10.3390/ani16091348 (PMC13163050; doi:10.3390/ani16091348)

Supplementary Material

Health-promoting phytobiotic-based feed additive improves skin and gill proteome response of infected fish

Elissavet A Arapi[1*](ORCID iD: 0000-0002-2187-786X)**, Laura Fernández-Alacid**[2] (ORCID iD: 0000-0001-7867-0985)**, Maria Mercè Isern-Subich**[3] (ORCID iD: 0000-0003-2048-0892)**, Waldo G. Nuez-Ortín**[3] (ORCID iD: 0000-0002-2991-1073)**, Antoni Ibarz**[2#] (ORCID iD: 0000-0002-1890-5305)**, Jo Cable**[1#] (ORCID iD: 0000-0002-8510-7055)

1 School of Biosciences and Water Research Institute, Cardiff University, Cardiff, CF10 3AX, UK.

2 Department of Cell Biology, Physiology and Immunology, Faculty of Biology, University of Barcelona, Avda. Diagonal 643, 08028, Barcelona, Spain.

3 Adisseo France S.A.S., Immeuble Antony Parc 2 10, Place du Général de Gaulle, 92160, Antony, France.

*Correspondance : Arapi Elissavet, E-mail : ArapiE@cardiff.ac.uk, 0044 (0)7472115621

# Joint last authors

Academic Editor: Firstname Lastname

Received: date

Revised: date

Accepted: date

Published: date

Citation: To be added by editorial staff during production.

Copyright: © 2025 by the authors. Submitted for possible open access publication under the terms and conditions of the Creative Commons Attribution (CC BY) license (https://creativecommons.org/licenses/by/4.0/).

Table S1. The 36 up-regulated proteins associated with the reactome pathway of immune system (HSA-168256; fdr=5.88e-05) in gill tissue of *Gyrodactylus turnbulli* infected susceptible guppies on Day 13 post-infection (Genecards, 2021; UniProt, 2021). See Figure 3 in Main text.

Protein	Protein name	Accession No.	Function
AKT1	Proline-rich AKT1 substrate 1	A0A3P9MVG3	Part of regulation of apoptotic process
APP	Amyloid-beta precursor protein	A0A3P9NS23	Functions as a cell surface receptor and performs physiological functions on the surface of neurons relevant to neurite growth, neuronal adhesion and axonogenesis
ATP6V0A1	V-type proton ATPase 116 kDa subunit a 1	A0A3P9Q1A9	A multisubunit enzyme that transports protons across cellular membranes
ATP8A1	Phospholipid-transporting ATPase IA	A0A3P9P581	Catalytic component of a P4-ATPase flippase complex
BPI	Bactericidal permeability-increasing protein	A0A3P9PV07	The cytotoxic action of BPI is limited to many species of Gram-negative bacteria
C1QB	Complement C1q subcomponent subunit B	A0A3P9NXV1	C1q is associated with the proenzymes C1r and C1s to yield C1, the first component of the serum complement system
C2	Complement component C2	A0A3P9NPC1	CPmponent C2 is part of the classical pathway of the complement system
C3	Complement component C3	A0A3P9MSY0	C3 plays a central role in the activation of the complement system
CASP8	Caspase-8	A0A3P9MZ09	Thiol protease that plays a key role in programmed cell death by acting as a molecular switch for apoptosis, necroptosis and pyroptosis
CD70	CD70 antigen	P32970	The ligand of the CD27 receptor, expressed at the surface of T cells
CD74	HLA class II histocompatibility antigen gamma chain	P04233	Plays a critical role in MHC class II antigen processing
CHIT1	Chitotriosidase-1	A0A3P9NAC8	Degrades chitin, chitotriose and chitobiose. May also participate in the defense against nematodes and other pathogens
CLEC4C	C-type lectin domain family 4 member C	A0A3P9PAC7	Lectin-type cell surface receptor which may play a role in antigen capturing by dendritic cells
COL1A1	Collagen alpha-1 (I) chain	A0A3P9NHJ7	Type I collagen is a member of group I collagen (fibrillar forming collagen)
COL1A2	Collagen alpha-2 (I) chain	A0A3P9NIL2	Type I collagen is a member of group I collagen (fibrillar forming collagen)
FBXO6	F-box only protein 6	A0A3P9N8T3	Involved in endoplasmic reticulum-associated degradation pathway for misfolded lumenal proteins
FCGR2A	Low affinity immunoglobulin gamma Fc region receptor II-a	A0A3P9N553	By binding to IgG it initiates cellular responses against pathogens and soluble antigens and promotes phagocytosis of opsonized antigens

GRB2	Growth factor receptor-bound protein 2	A0A3P9PAL6	Non-enzymatic adapter protein that plays a pivotal role in precisely regulated signalling cascades, including signalling transduction and gene expression
HLA-DRB1	HLA class II histocompatibility antigen, DRB1 beta chain	A0A3P9NYV4	A beta chain of antigen-presenting major histocompatibility complex class II (MHCII) molecule
ISG20	Interferon-stimulated gene 20 kDa protein	A0A3P9Q0G3	Exhibits antiviral activity against RNA viruses including hepatitis C virus (HCV), hepatitis A virus (HAV) and yellow fever virus (YFV)
KCMF1	E3 ubiquitin-protein ligase KCMF1	A0A3P9NWE4	E3 ubiquitin-protein ligase which accepts ubiquitin from an E2 ubiquitin-conjugating enzyme and then transfers it to targeted substrates
LBP	Lipopolysaccharide-binding protein	A0A3P9MXE7	Plays a role in the innate immune response
LGALS3	Galectin-3	A0A3P9P5J9	Part of antimicrobial humoral immune response mediated by antimicrobial peptide and monocyte, eosinophil, macrophage chemotaxis
LIMK1	LIM domain kinase 1	A0A3P9QEM7	Plays an essential role in the regulation of actin filament dynamics
MME	Membrane metallo-endopeptidase-like 1	A0A3P9NKH0	Part of protein processing and proteolysis
N4BP1	NEDD4-binding protein 1	A0A3P9NWI2	Potent suppressor of cytokine production that acts as a regulator of innate immune signalling and inflammation
NLRP1	NACHT, LRR and PYD domains-containing protein 1	A0A3P9P866	Sensor component of the NLRP1 inflammasome, which mediates inflammasome activation in response to various pathogen-associated signals, leading to subsequent pyroptosis
PRDX6	Peroxiredoxin-6	A0A3P9MZF5	Plays role in cell protection against oxidative stress
PTK2	Focal adhesion kinase 1	A0A3P9MVQ2	Non-receptor kinase that plays an essential role in ell cycle progression, cell proliferation and apoptosis
PTPN1	Tyrosine-protein phosphatase non-receptor type 1	A0A3P9P4Z3	Acts as regulator of endoplasmic reticulum unfolded protein response
S100A1	Protein S100-A1	A0A3P9NP99	Small calcium binding protein that plays important roles Ca^{2+} homeostasis, chondrocyte biology and cardiomyocyte regulation
SAMHD1	Deoxynucleoside triphosphate triphosphohydrolase SAMHD1	A0A3P9PZX9	Protein acts as a host restriction factor involved in defense response to virus
SIGLEC14	Sialic acid-binding Ig-like lectin 14	A0A3P9N8T5	Sialic acid-binding paired receptor which may activate associated receptors
TIMP2	Metalloproteinase inhibitor 2	A0A3P9N7J3	Complexes with metalloproteinases (such as collagenases)

TP53	Cellular tumour antigen p53	A0A3P9PYT5	Multifunctional transcription factor that induces cell cycle arrest, DNA repair or apoptosis
TRIM25	E3 ubiquitin/ISG15 ligase TRIM25	A0A3P9MS71	Involved in innate immune defense against viruses

Table S2. The 15 down-regulated proteins associated with the reactome pathway of neutrophil degranulation (HSA-6798695; fdr=1.63e-21) in susceptible gill tissue of *Gyrodactylus turnbulli* infected guppies on Day 13 post-infection (Genecards, 2021; UniProt, 2021). See Figure 4.

Protein	Protein Name	Accession No.	Function
AHSG	Alpha-2-HS-glycoprotein	A0A3P9NGJ5	Promotes endocytosis
ATP8A1	Phospholipid-transporting ATPase		Plays a direct role in the translocation of protons across the membrane
B2M	Beta-2-microglobulin	A0A3P9PWJ4	Component of the class I major histocompatibility complex (MHC) involved in the presentation of peptide antigens to the immune system
CHIT1	Chitotriosidase-1	A0A3P9NAC8	Degrades chitin, chitotriose and chitobiose. May also participate in the defense against nematodes and other pathogens
CSNK2B	Casein kinase II subunit beta	A0A3P9PJ41	Regulatory subunit of casein kinase II/CK2
CYBB	Cytochrome b-245 heavy chain	A0A3P9PJE7	Part of inflammatory and innate immune response
DNAJC5	DnaJ homolog subfamily C member 5	A0A3P9Q6G8	Acts as a general chaperone in regulated exocytosis
DSC1	Desmocollin-1	A0A3P9NCN5	Component of desmosome cell-cell junctions required for positive regulation of cellular adhesion
ELANE	Neutrophil elastase	A0A3P9PP37	Modifies the functions of natural killer cells, monocytes and granulocytes
HBB	Haemoglobin subunit beta	A0A3P9NNH2	Involved in oxygen transport from the lung to the various peripheral tissues
HSP90AA1	Heat shock protein HSP 90-alpha	A0A3P9PGJ0	Promotes the maturation, structural maintenance and proper regulation of specific target proteins involved for instance in cell cycle control and signal transduction
KRT1	Keratin, type II cytoskeletal 1	P04264	Involved in the activity of kinases.
LGALS3	Galectin-3	A0A3P9N4Q8	Required for differentiation of columnar epithelial cells during embryogenesis
MAPK1	Mitogen-activated protein kinase 1	A0A3P9NB64	Part of apoptotic process
OLFM4	Olfactomedin-4	A0A3P9N2V9	Facilitates cell adhesion, most probably through interaction with cell surface lectins and cadherin

Table S3. The 17 up-regulated proteins associated with the biological response of programmed cell death (GO:0012501; fdr=0.020) in skin tissue of responding *Gyrodactylus turnbulli* infected guppies on Day 13 post-infection (Genecards, 2021; UniProt, 2021). See Figure 5.

Protein	Protein Name	Accession No.	Function
AKT1	Proline-rich AKT1 substrate 1	A0A3P9MVG3	Part of regulation of apoptotic process
BRCA1	Breast cancer type 1 susceptibility protein	A0A3P9NIS4	Plays a role in intrinsic apoptotic signalling pathway in response to DNA damage
FOXO1	Forkhead box protein O1	A0A3P9QHL8	Promotes neural cell death
H1F0	Histone H1.0	A0A3P9NJ60	Found in cells that are in terminal stages of differentiation or that have low rates of cell division
HMGB2	High mobility group protein B2	A0A3P9N879	Involved in inflammatory response to antigenic stimulus coupled with proinflammatory activity
INPP5D	Phosphatidylinositol 3,4,5-trisphosphate 5-phosphatase 1	A0A3P9NXA4	Integrated in negative regulation of immune response, monocyte and neutrophil differentiation and in positive regulation of apoptotic process
KRT14	Keratin, type I cytoskeletal 14	P02533	Enhances the mechanical properties involved in resilience of keratin
KRT17	Keratin, type I cytoskeletal 17	Q04695	Involved in tissue repair, acts as a promoter of epithelial proliferation by acting a regulator of immune response in skin and may act as an autoantigen
KRT18	Keratin, type I cytoskeletal 18	A0A3P9PAG9	Involved in intermediate filament cytoskeleton organization
KRT19	Keratin, type I cytoskeletal 19	A0A3P9MTP9	Part of extrinsic apoptotic signalling pathway
KRT4	Keratin, type II cytoskeletal 4	P07744	Part of negative regulation of epithelial cell proliferation
KRT6A	Keratin, type II cytoskeletal 6A	P02538	Epidermis-specific type I keratin involved in wound healing
KRT6B	Keratin, type II cytoskeletal 6B	P04259	Plays a role in structural constituent of cytoskeleton
MAP3K5	Mitogen-activated protein kinase 5	A0A3P9PSE5	Part of the apoptotic signalling pathway
MX1	Interferon-induced GTP-binding protein Mx1	A0A3P9Q1X8	Interferon-induced dynamin-like GTPase with antiviral activity against a wide range of RNA viruses and some DNA viruses
PRKCA	Protein kinase C alpha type	A0A3P9NIW3	Part of the apoptotic signalling pathway
YARS	Tyrosine--tRNA ligase, cytoplasmic	A0A3P9P5E9	Part of the apoptotic process

Table S4. The 24 down-regulated proteins associated with the reactome pathway of innate immune system (HSA-168249; fdr<0.0001) in skin tissue of responding *Gyrodactylus turnbulli* infected guppies on Day 13 post-infection (Genecards, 2021; UniProt, 2021). See Figure 6.

Protein	Protein name	Accession No.	Function
AHSG	Alpha-2-HS-glycoprotein	A0A3P9NGJ5	Promotes endocytosis
ANPEP	Aminopeptidase N	A0A3P9N231	Part of peptide binding and signalling receptor activity
AOC1	Amiloride-sensitive amine oxidase/copper containing	A0A3P9PPK7	Catalyses the degradation of compounds involved in allergic and immune responses cell proliferation, tissue differentiation, tumour formation, and possibly apoptosis
APRT	Adenine phosphoribosyltransferase	A0A3P9NUB7	Catalyses a salvage reaction resulting in the formation of AMP
ATP6V1E1	V-type proton ATPase subunit E1	A0A3P9Q940	Part of regulation of the macroautophagy process
C1QC	Complement C1q subcomponent subunit C	A0A3P9QI46	Associated with the proenzymes C1r and C1s to yield C1, the first component of the serum complement system
C5	Complement component C5a	A0A3P9N4W2	Part of complement activation, inflammatory response and innate immune response
CD209	CD209 antigen	A0A3P9MSF4	Pathogen-recognition receptor and involved in initiation of primary immune response
CD59	CD59 molecule – blood group	A0A3P9P292	Part of defence response to bacteria
CNPY3	Protein canopy homolog 3	A0A3P9P992	Part of the innate immune response
HLA-A	H-2 class I histocompatibility antigen, K-K alpha chain-like	A0A3P9P4Q3	Antigen-presenting major histocompatibility complex class I (MHCI) molecule, integral part of innate immune response
HSP90B1	Endoplasmin	A0A3P9P4A8	Molecular chaperone that functions in the processing and transport of secreted proteins
IQGAP1	Ras GTPase-activating	A0A3P9N7K1	Part of the negative regulation of apoptotic process
ITGAM	Integrin alpha-M	A0A3P9QJ70	Implicated in various adhesive interactions of monocytes, macrophages and granulocytes as well as in mediating the uptake of complement-coated particles and pathogens
LGALS3	Galectin-3	A0A3P9P5J9	Part of antimicrobial humoral immune response mediated by antimicrobial peptide and monocyte, eosinophil, macrophage chemotaxis
LYZ	Lysozyme C	A0A3P9PX78	Has a bacteriolytic function, enhances the activity of immunoagents and associated with the monocyte-macrophage system

NCF2	Neutrophil cytosol factor 2	A0A3P9NVF4	Part of phagocytosis, cellular defence and innate immune response
PDAP1	28 kDa heat- and acid-stable phosphoprotein	A0A3P9PUD0	Plays a role in signal transduction
PRSS3	Trypsin-3	A0A3P9P484	Part of antimicrobial humoral response
PTX3	Pentraxin-related protein PTX3	A0A3P9Q3A3	Plays a role in the regulation of innate resistance to pathogens, inflammatory reactions, possibly clearance of self-components
RAB10	Ras-related protein	A0A3P9PPQ1	Regulator of TLR4 transport, a toll-like receptor to the plasma membrane and therefore may be important for innate immune response
RAB5C	Ras-related protein	A0A3P9QF24	Involved in vesicular traffic and protein transport
TRAPPC1	Trafficking protein particle complex subunit 1	A0A3P9NST9	Plays a role in vesicular transport from endoplasmic reticulum to Golgi
TUBB	Tubulin beta chain	A0A3P9NUB9	Involved in protein domain specific binding

Table S5. The 26 up-regulated proteins associated with the reactome pathway of immune response (HAS-168256; fdr=3.02e-23) in skin tissue of responding *Gyrodactylus turnbulli* infected guppies on Day 17 post-infection (Genecards, 2021; UniProt, 2021). See Figure 7.

Protein	Protein name	Accession No.	Function
AHSG	Alpha-2-HS-glycoprotein	P12763	Promotes endocytosis
ANPEP	Aminopeptidase N	A0A3P9N231	Part of peptide binding and signalling receptor activity
ANXA1	Annexin A1	A0A3P9NLV1	Plays important roles in the innate immune response and anti-inflammatory activity
ATP6V0D1	V-type proton ATPase subunit d1	A0A3P9NSY7	Part of multisubunit enzyme composed of a peripheral complex (V1) that translocate protons
ATP6V0E1	V-type proton ATPase subunit e 1	A0A3P9QI23	Part of multisubunit enzyme composed of a peripheral complex (V1) that translocate protons
CA1	Carbonic anhydrase 1	A0A3P9NPT1	Reversible hydration of carbon dioxide
CD74	HLA class II histocompatibility antigen gamma chain	A0A3P9N5V7	Plays a critical role in MHC class II antigen processing
CTSD	Cathepsin D	A0A3P9Q0W4	Acid protease active in intracellular protein breakdown
CTSH	Pro-cathepsin H	A0A3P9NNY2	Important for the overall degradation of proteins in lysosomes
CTSS	Cathepsin S	A0A3P9Q4E4	Responsible for the removal of the invariant chain from MHC class II molecules and MHC class II antigen presentation

DOCK1	Dedicator of cytokinesis protein 1	A0A3P9NIS5	Involved in cytoskeletal rearrangements required for phagocytosis of apoptotic cells and cell motility
HLA-A	H-2 class I histocompatibility antigen, K-K alpha chain-like	A0A3P9P4Q3	Antigen-presenting major histocompatibility complex class I (MHCI) molecule, integral part of innate immune response
HLA-DPA1	HLA class II histocompatibility antigen, DP alpha 1 chain	A0A3P9NYT7	Part of adaptive immune response and antigen processing
HLA-DRB1	HLA class II histocompatibility antigen, DRB1 beta chain	A0A3P9NYV4	Part of adaptive immune response and antigen processing
ITGAL	Integrin alpha-L	A0A3P9N5L6	Associated with ICAM3, contributes to apoptotic neutrophil phagocytosis by macrophages
LYZ	Lysozyme C	A0A3P9PX78	Has a bacteriolytic function, enhances the activity of immunoagents and associated with the monocyte-macrophage system
MRC1	Macrophage mannose receptor 1	A0A3P9PFL5	Acts as a phagocytic receptor for bacteria, fungi and other pathogens
NLRP1	NACHT, LRR and PYD domains-containing protein 1	A0A3P9NM53	Sensor component of the NLRP1 inflammasome, which mediates inflammasome activation in response to various pathogen-associated signals, leading to subsequent pyroptosis
NUP43	Nucleoporin Nup43	A0A3P9PPI5	Component of the Nup107-160 subcomplex of the nuclear pore complex (NPC)
PIN1	Peptidyl-prolyl cis-trans isomerase NIMA-interacting 1	A0A3P9Q7U3	Catalyses cis-trans isomerization of phosphorylated phosphoglycerate kinase PGK1
PSMB1	Proteasome subunit beta type-1	A0A3P9NZV3	Component of the 20S core proteasome complex involved in the proteolytic degradation of most intracellular proteins
PTK2	Focal adhesion kinase 1	A0A3P9PAB2	Non-receptor kinase that plays an essential role in ell cycle progression, cell proliferation and apoptosis
PVR	Poliovirus receptor	A0A3P9MV83	Plays a role in mediating tumor cell invasion and migration
TAPBP	Tapasin	A0A3P9N225	Involved in the association of MHC class I with transporter associated with antigen processing (TAP)
TUBB	Tubulin beta chain	A0A3P9NUB9	Involved in protein domain specific binding
UBAC1	Ubiquitin-associated domain-containing protein 1	A0A3P9NDB1	E3 ubiquitin-protein ligase complex that mediates polyubiquitination of target proteins

Table S6. The 28 up-regulated proteins associated with the reactome pathway of immune response (HAS-168256; fdr=3.02e-23) in resistant *Gyrodactylus turnbulli* infected guppies on Day 13 post-infection (Genecards, 2021; UniProt, 2021). See Figure 8.

Protein	Protein name	Accession No.	Function
AOC1	Amiloride-sensitive amine oxidase/copper containing	A0A3P9NJ58	Catalyses the degradation of compounds involved in allergic and immune responses cell proliferation, tissue differentiation, tumour formation, and possibly apoptosis
ATP6V1A	V-type proton ATPase catalytic subunit A	A0A3P9N1F0	Biological function includes regulation of macroautophagy
C1QB	Complement C1q subcomponent subunit B	A0A3P9QI83	C1q is associated with the proenzymes C1r and C1s to yield C1, the first component of the serum complement system
C3	Complement component C3	A0A3P9MSY0	C3 plays a central role in the activation of the complement system
C4B	Complement C4-B	A0A3P9N1S6	Non-enzymatic component of the C3 and C5 convertases and thus essential for the propagation of the classical complement pathway
C5	Complement component C5a	A0A3P9N4W2	Part of complement activation, inflammatory response and innate immune response
C7	Complement component C7	A0A3P9MX95	Plays a key role in the innate and adaptive immune response by forming pores in the plasma membrane of target cells. C7 serves as a membrane anchor.
CA1	Carbonic anhydrase 1	A0A3P9NPT1	Reversible hydration of carbon dioxide
CFD	Complement factor D	A0A3P9NII0	Part of complement activation
CFHR1	Complement factor H-related protein 1	A0A3P9NG87	Involved in complement regulation
CFI	Complement factor I	A0A3P9PJG0	Part of complement activation and innate immune response
COL2A1	Collagen alpha-1(II) chain	A0A3P9NNJ3	Essential for the ability of cartilage to resist compressive forces.
CRISPLD2	Cysteine-rich secretory protein LCCL domain-containing 2	A0A3P9QJF0	Involved in extracellular matrix organization
CTSB	Cathepsin B	A0A3P9NNC9	Part of apoptotic process regulation
CYBB	Cytochrome b-245 heavy chain	A0A3P9PJE7	Part of inflammatory and innate immune response
HLA-A	H-2 class I histocompatibility antigen, K-K alpha chain-like	A0A3P9P4Q3	Major histocompatibility complex class I (MHCI) molecule, integral part of innate immune response
HLA-DPA1	HLA class II histocompatibility antigen, DP alpha 1 chain	A0A3P9NYT7	Part of adaptive immune response and antigen processing

IGF2R	Cation-independent mannose-6-phosphate receptor	A0A3P9PYC3	Acts as a positive regulator of T-cell coactivation
ITGAL	Integrin alpha-L	A0A3P9N5L6	Associated with ICAM3, contributes to apoptotic neutrophil phagocytosis by macrophages
LAMA5	Laminin subunit alpha-5	A0A3P9Q7N7	Part of extracellular matrix structure
MAVS	Mitochondrial antiviral signalling protein	A0A3P9NLD2	Required for innate immune defence against viruses and bacteria
MME	Membrane metallo-endopeptidase-like 1	A0A3P9NKH0	Part of protein processing and proteolysis
PRCP	Lysosomal Pro-X carboxypeptidase	A0A3P9PJ92	Part of proteolysis
PRSS3	Trypsin-3	A0A3P9NPW3	Part of antimicrobial humoral response
SYNGR1	Synaptogyrin-1	A0A3P9N1G8	Plays a role in regulated exocytosis
TBK1	Serine/threonine-protein kinase TBK1	A0A3P9PAZ0	Part of inflammatory, defence and innate immune response
TUBA1A	Tubulin alpha-1A chain	A0A3P9MWX7	Structural constituent of cytoskeleton
WAS	WASP homolog-associated protein with actin	A0A3P9N424	Part of actin filament organization

Table S7. The 16 down-regulated proteins associated with cell death processes (GO:0008219; fdr=0.0277), in skin tissue of resistant *Gyrodactylus turnbulli* infected guppies on Day 13 post-infection (Genecards, 2021; UniProt, 2021). See Figure 9.

Protein	Protein name	Accession No.	Function
CAPNS1	Calpain small subunit 1	A0A3P9NIW9	Plays a role in positive regulation of cell population proliferation and macroautophagy
DNASE1	Deoxyribonuclease-1	A0A3P9QI02	Part of apoptotic process, neutrophil activation involved in immune response, and regulation of acute inflammatory response
EVPL	Envoplakin	A0A3P9Q327	Component of the cornified envelope of keratinocytes, involved in epidermis development and wound healing
GAPDH	Glyceraldehyde-3-phosphate dehydrogenase	A0A3P9MW16	Involved in antimicrobial humoral immune response, defence response to fungus, killing of cells of other organisms and regulation of macroautophagy
KRT13	Keratin, type I cytoskeletal 13	A0A3P9N825	Plays a role in cytoskeleton organization

KRT16	Keratin, type I cytoskeletal 16	P08779	Plays a key role in skin, acts as a regulator of innate immunity in response to skin barrier breach: required for some inflammatory checkpoint for the skin barrier maintenance
KRT17	Keratin, type I cytoskeletal 17	Q04695	Involved in tissue repair, acts as a promoter of epithelial proliferation by acting a regulator of immune response
KRT34	Keratin, type I cuticular Ha4	O76011	Involved in epidermis development
KRT8	Keratin, type II cytoskeletal 8	A0A3P9MYE0	Part of extrinsic apoptotic signalling pathway
MAPK1	Mitogen-activated protein kinase 1	A0A3P9QD90	Part of apoptotic process
PARP1	Poly [ADP-ribose] polymerase 1	A0A3P9MWA6	Part of apoptotic process
PMP22	Peripheral myelin protein 22	A0A3P9N6T8	Part of cell death
PPL	Periplakin	A0A3P9P5K6	Component of the cornified envelope of keratinocytes
SLC25A4	ADP/ATP translocase 1	A0A3P9ND30	Part of negative regulation of necroptotic process
TGM1	Protein-glutamine gamma-glutamyltransferase K	A0A3P9Q8A0	Plays a role in keratinization
TRIM35	E3 ubiquitin-protein ligase	A0A3P9QHX6	Positive regulation of apoptotic process

Table S8. The 38 down-regulated proteins in resistant fish gills associated with the reactome pathway of immune response (HSA-168256; fdr=0.0027) in *Gyrodactylus turnbulli* infected guppies on Day 13 post-infection (Genecards, 2021; UniProt, 2021). See Figure 10.

Protein	Protein name	Accession No.	Function
ACTB	Actin, cytoplasmic 1	A0A3P9PT13	Actin polymerizes to produce filaments forming cross-linked networks in the cell cytoplasm
AKT1	Proline-rich AKT1 substrate 1	A0A3P9MVG3	Part of regulation of apoptotic process
ATP5MC3	ATP synthase F (0) complex subunit C3,	P48201	Mitochondrial membrane ATP synthase
CA1	Carbonic anhydrase 1	A0A3P9NPT1	Reversible hydration of carbon dioxide
CD209	CD209 antigen	A0A3P9MSF4	Pathogen-recognition receptor and involved in initiation of primary immune response
CD34	Hematopoietic progenitor cell antigen CD34	A0A3P9PE04	Possible adhesion molecule mediating the attachment of stem cells to the bone marrow extracellular matrix or directly to stromal cells
COL1A1	Collagen alpha-1 (I) chain	A0A3P9NHJ7	Type I collagen is a member of group I collagen (fibrillar forming collagen)
COL1A2	Collagen alpha-1(I) chain	A0A3P9NIL2	Essential for the ability of cartilage to resist compressive forces

COL2A1	Collagen alpha-1(II) chain	A0A3P9NNJ3	Essential for the ability of cartilage to resist compressive forces
COTL1	Coactosin-like protein	A0A3P9MUH8	Influencing both stability and activity in leukotrienes synthesis
CPNE3	Copine-3	A0A3P9NK77	Plays a role in ERBB2-mediated tumor cell migration
CREBBP	CREB-binding protein	A0A3P9PCZ1	Acetylates histones, giving a specific tag for transcriptional activation
CXADR	Coxsackievirus and adenovirus receptor	A0A3P9NXV6	Involved in proliferation and production of cytokines and growth factors by T-cells that in turn stimulate epithelial tissues repair.
FGB	Fibrinogen beta chain	A0A3P9NQP0	Facilitate the antibacterial immune response via both innate and T-cell mediated pathways
FGG	Fibrinogen gamma chain	A0A3P9N2D2	Facilitate the antibacterial immune response via both innate and T-cell mediated pathways
FOXO3	Forkhead box protein O3	A0A3P9PSD1	Key regulator of regulatory T-cells (Treg) differentiation
IFI30	Gamma-interferon-inducible lysosomal thiol reductase	A0A3P9QF45	Plays an important role in antigen processing
INPP5D	inositol polyphosphate-5-phosphatase D	Q92835	Key regulator of neutrophil migration, by governing the formation of the leading edge and polarization required for chemotaxis
IRS1	Insulin receptor substrate 1	A0A3P9P8R6	Important role in development, growth, glucose homeostasis as well as lipid metabolism
ITCH	E3 ubiquitin-protein ligase Itchy homolog	A0A3P9PUR6	Acts as an E3 ubiquitin-protein ligase
ITGB1	Integrin beta-1	A0A3P9PXA0	Present in oocytes and is involved in sperm-egg fusion
KRT1	Keratin, type II cytoskeletal 1	P04264	Involved in the activity of kinases.
LYZ	Lysozyme C	A0A3P9PX78	Bacteriolytic function, enhances the activity of immunoagents and associated with the monocyte-macrophage system
MUC2	Mucin-2	A0A3P9PDF7	Provides a protective, lubricating barrier against particles and infectious agents at mucosal surfaces
MX1	Interferon-induced GTP-binding protein Mx1	A0A3P9Q1X8	Interferon-induced dynamin-like GTPase with antiviral activity against a wide range of RNA viruses and some DNA viruses
NLRC3	NLR family CARD domain-containing protein 3	A0A3P9QEY9	Negative regulator of the innate immune response
PECAM1	Platelet endothelial cell adhesion molecule	A0A3P9PNL9	Required for leukocyte transendothelial migration (TEM) under most inflammatory conditions
PRSS3	Trypsin-3	A0A3P9NPW3	Part of antimicrobial humoral response
PTK2	Focal adhesion kinase 1	A0A3P9PYG1	Non-receptor kinase that plays an essential role in ell cycle progression, cell proliferation and apoptosis
PTX3	Pentraxin-related protein PTX3	A0A3P9Q3A3	Plays a role in the regulation of innate resistance to pathogens, inflammatory

			reactions, possibly clearance of self-components
RAB18	Ras-related protein Rab -18	A0A3P9N3F0	Key regulator of intracellular membrane trafficking, from the formation of transport vesicles to their fusion with membranes
RICTOR	Rapamycin-insensitive companion of mTOR	A0A3P9NZU8	Component of rapamycin complex 2 (mTORC2), transducing signals from growth factors to pathways involved in proliferation, cytoskeletal organization, lipogenesis and anabolic output
S100A1	Protein S100-A1	A0A3P9Q4K6	Small calcium binding protein that plays important roles Ca^{2+} homeostasis, chondrocyte biology and cardiomyocyte regulation
SEC61G	Protein transport protein Sec61 subunit gamma	A0A3P9PM20	Component of SEC61 channel-forming translocon complex mediating transport of signal peptide-containing polypeptides across the endoplasmic reticulum
SOD1	Superoxide dismutase [Cu-Zn] B	A0A3P9PA62	Destroys radicals which are normally produced within the cells, and which are toxic to biological systems
TIMP2	Metalloproteinase inhibitor 2	A0A3P9N7J3	Complexes with metalloproteinases (such as collagenases)
TXN	Thioredoxin	A0A3P9N9J4	Participates in various redox reactions
UBE2N	Ubiquitin-conjugating enzyme E2 N	A0A3P9PXH7	Mediates transcriptional activation of target genes and plays a role in the control of progress through the cell cycle and differentiation

Table S9. The 17 up-regulated proteins in resistant fish skin in *Gyrodactylus turnbulli* infected guppies on Day 17 post-infection, associated with the reactome pathways of innate immune system (HSA-168249; fdr=5.13e-19; Genecards, 2021; UniProt, 2021). See Figure 11.

Protein	Protein name	Accession No.	Function
APRT	Adenine phosphoribosyltransferase	A0A3P9NUB7	Catalyses a salvage reaction resulting in the formation of AMP, that is energetically less costly than de novo synthesis
CD209	CD209 antigen	A0A3P9MSF4	Pathogen-recognition receptor and involved in initiation of primary immune response
CDC42	Cell division control protein 42 homolog	A0A3P9N8S0	Plays a role in phagocytosis through organization of the F-actin cytoskeleton and formation of phagocytic cups
CKAP4	Cytoskeleton-associated protein 4	A0A3P9QJS7	Mediates the anchoring of the endoplasmic reticulum to microtubules
DNAJC13	DnaJ homolog subfamily C member 13	A0A3P9NDK2	Involved in membrane trafficking through early endosomes

GRB2	Growth factor receptor-bound protein 2	A0A3P9PAL6	Non-enzymatic adapter protein that plays a pivotal role in precisely regulated signalling cascades, including signalling transduction and gene expression
LYZ	Lysozyme C	A0A3P9PX78	Bacteriolytic function, enhancing activity of immunoagents and associated with the monocyte-macrophage system
MAPK1	Mitogen-activated protein kinase 1	A0A3P9QD90	Part of apoptotic process
MME	Membrane metallo-endopeptidase-like 1	A0A3P9NKH0	Part of protein processing and proteolysis
NCF2	Neutrophil cytosol factor 2	A0A3P9NVF4	Part of phagocytosis, cellular defence and innate immune response
PLA2G2A	Phospholipase A2	A0A3P9NV32	Induces cell proliferation in an integrin-dependent manner
PPIA	Peptidyl-prolyl cis-trans isomerase A	A0A3P9MWA7	Exerts a strong chemotactic effect on leukocytes
PSMB1	Proteasome subunit beta type-1	A0A3P9NAM2	Component of the 20S core proteasome complex involved in the proteolytic degradation of most intracellular proteins
RAB5C	Ras-related protein	A0A3P9QF24	Involved in vesicular traffic and protein transport
SUGT1	Protein SGT1 homolog	A0A3P9MZC3	May play a role in ubiquitination and subsequent proteasomal degradation of target proteins
SURF4	Surfeit locus protein 4	A0A3P9PLP6	Plays a role in the maintenance of the architecture of the endoplasmic reticulum-Golgi intermediate compartment and of the Golgi
TXNDC5	Thioredoxin domain-containing protein 5	A0A3P9PV76	Involved in the formation of disulfide bonds in proteins. Can reduce insulin disulfide bonds

Figure S1. Interactomes of proteins differentially expressed in skin tissue of *Gyrodactylus* infected guppies (*Poecilia reticulata*). A. Interactome of the 351 proteins up-regulated in the guppies provided with the Apex supplement over the control diet on Day 13 post-infection (number of nodes: 262, number of edges: 594, avg. local clustering coefficient: 0.415, PPI enrichment p<1.0e-16). **B.** Interactome of the 484 proteins down-regulated in the guppies provided with the Apex supplement over the control diet on Day 13 post-infection (number of nodes: 375, number of edges: 1460, avg. local clustering coefficient: 0.404, PPI enrichment p<1.0e-16). **C**. Interactome of the 242 proteins up-regulated in the guppies provided with the Apex supplement over the control diet on Day 17 post-infection (number of nodes: 187, number of edges: 364, avg. local clustering coefficient: 0.364, PPI enrichment p=3.89e-08). **D**. Interactome of the 245 proteins down-regulated in the guppies provided with the Apex supplement over the control diet on Day 17 post-infection (number of nodes: 198, number of edges: 331, avg. local clustering coefficient: 0.407, PPI enrichment p=3.78e-05).

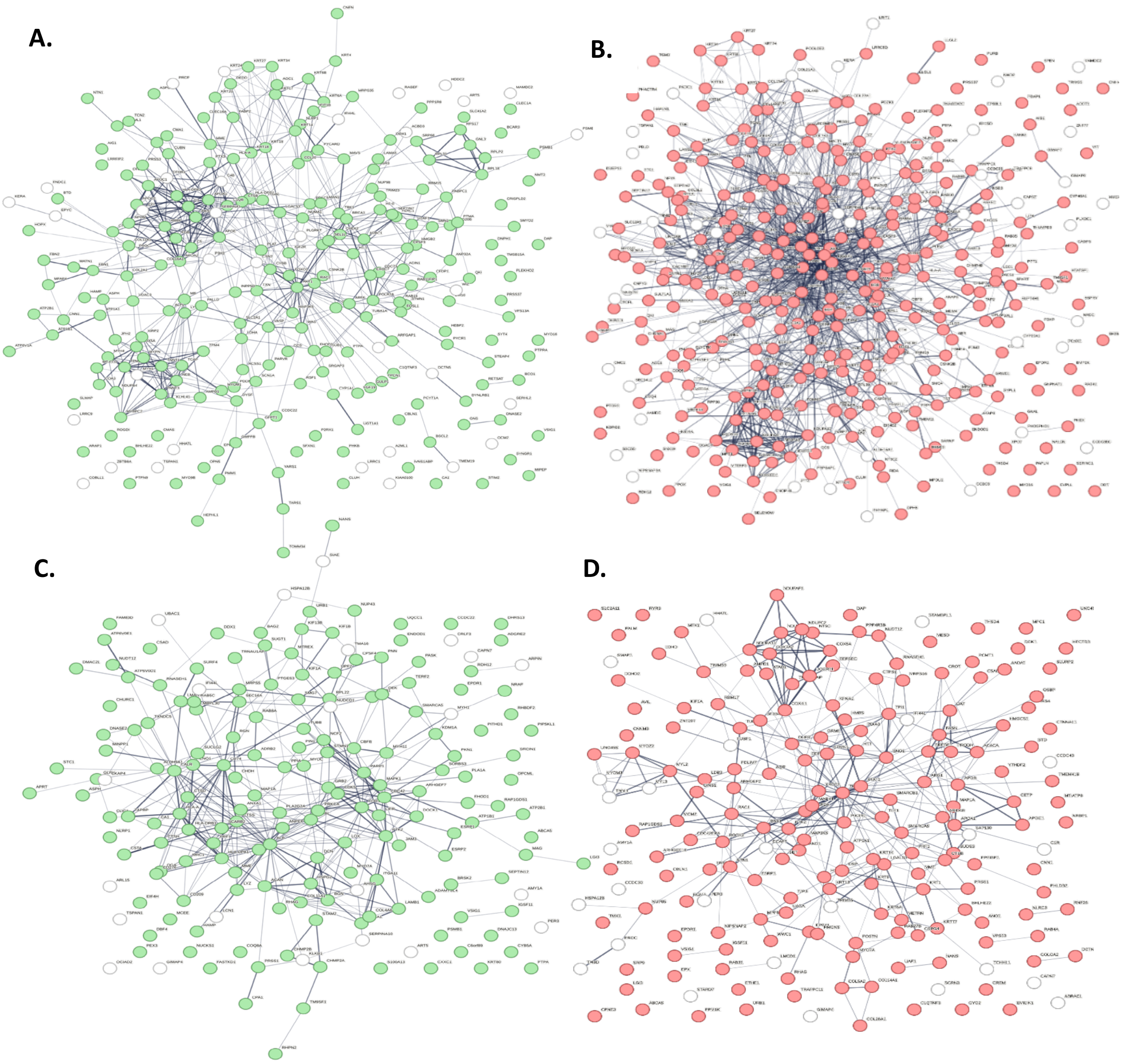

Figure S2. Interactomes of proteins differentially expressed in gill tissue of *Gyrodactylus* infected guppies (*Poecilia reticulata*). A. Interactome of the 427 proteins up-regulated in the guppies provided with the Apex supplement over the control diet on Day 13 post-infection (number of nodes: 323, number of edges: 908, avg. local clustering coefficient: 0.386, PPI enrichment p=1.56e-09). **B.** Interactome of the 785 proteins down-regulated in the guppies provided with the Apex supplement over the control diet on Day 13 post-infection (number of nodes: 532, number of edges: 2785, avg. local clustering coefficient: 0.405, PPI enrichment p<1.0e-16). **C.** Interactome of the 507 proteins up-regulated in the guppies provided with the Apex supplement over the control diet on Day 17 post-infection (number of nodes: 372, number of edges: 1038, avg. local clustering coefficient: 0.368, PPI enrichment p=4.97e-10). **D.** Interactome of the 480 proteins down-regulated in the guppies provided with the Apex supplement over the control diet on Day 17 post-infection (number of nodes: 381, number of edges: 1004, avg. local clustering coefficient: 0.4, PPI enrichment p=1.11e-16).

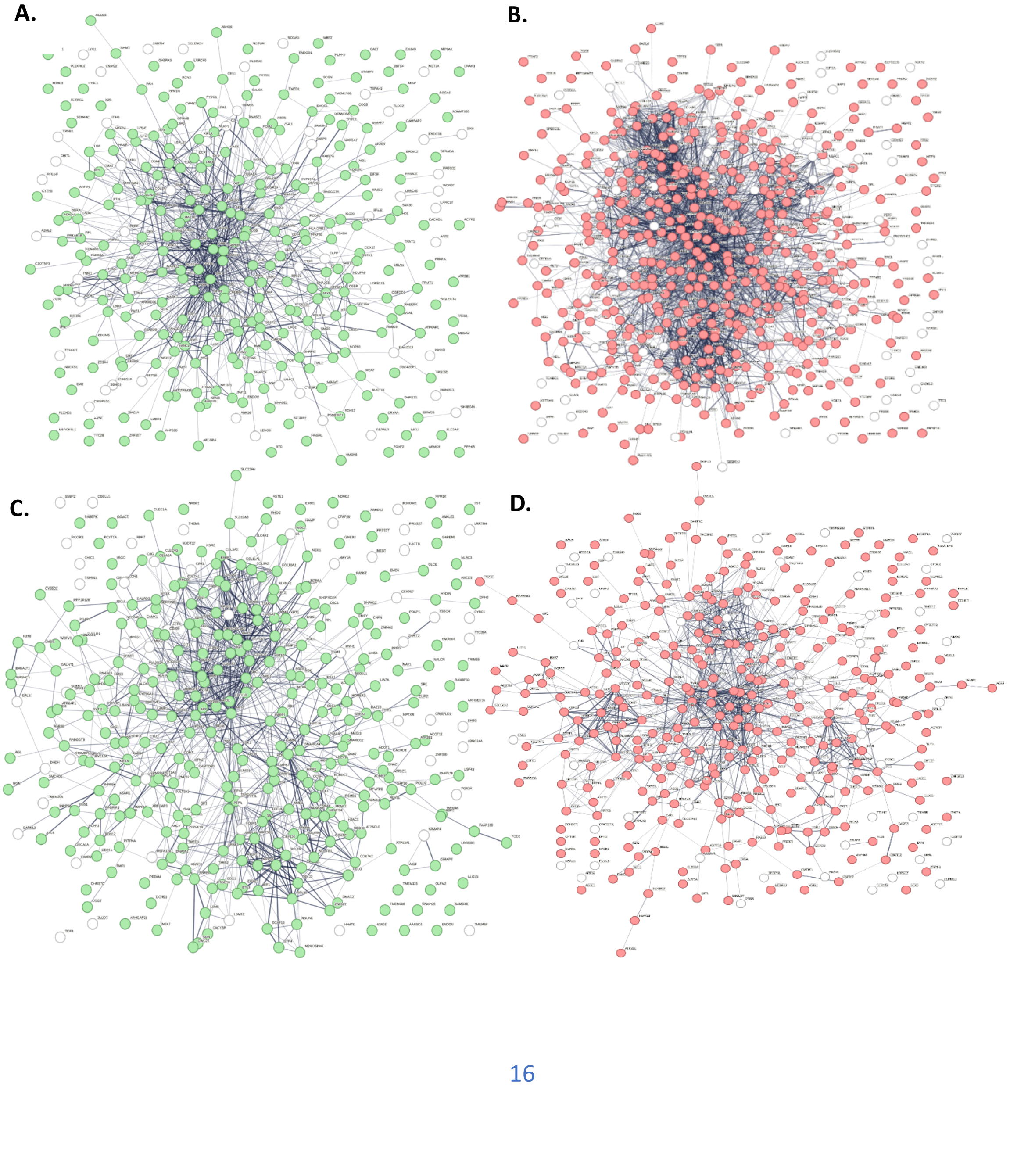

Figure S3. Interactomes of proteins differentially expressed in skin tissue of *Gyrodactylus* infected guppies (*Poecilia reticulata*) on Day 13 post-infection. A. Interactome of the 86 proteins up-regulated in the susceptible guppies provided with the Apex® Branchia supplement over the control diet (number of nodes: 74, number of edges: 33, avg. local clustering coefficient: 0.334, PPI enrichment p=0.0649). **B.** Interactome of the 164 proteins down-regulated in the susceptible guppies provided with the Apex® Branchia supplement over the control diet (number of nodes: 152, number of edges: 202, avg. local clustering coefficient: 0.358, PPI enrichment p=2.09e-06). **C.** Interactome of the 123 proteins up- regulated in the responding guppies provided with the Apex® Branchia supplement over the control diet. **D.** Interactome of the 218 proteins down-regulated in the responding guppies provided with the Apex® Branchia supplement over the control diet (number of nodes: 189, number of edges: 469, avg. local clustering coefficient: 0.448, PPI enrichment p<1.0e-16). **E.** Interactome of the 142 proteins up- regulated in the resistant guppies provided with the Apex® Branchia supplement over the control diet (number of nodes: 123, number of edges: 128, avg. local clustering coefficient: 0.367, PPI enrichment p=5.92e-12). **F.** Interactome of the 102 proteins down-regulated in the resistant guppies provided with the Apex® Branchia supplement over the control diet (number of nodes: 91, number of edges: 113, avg. local clustering coefficient: 0.388, PPI enrichment p=2.21e-08).

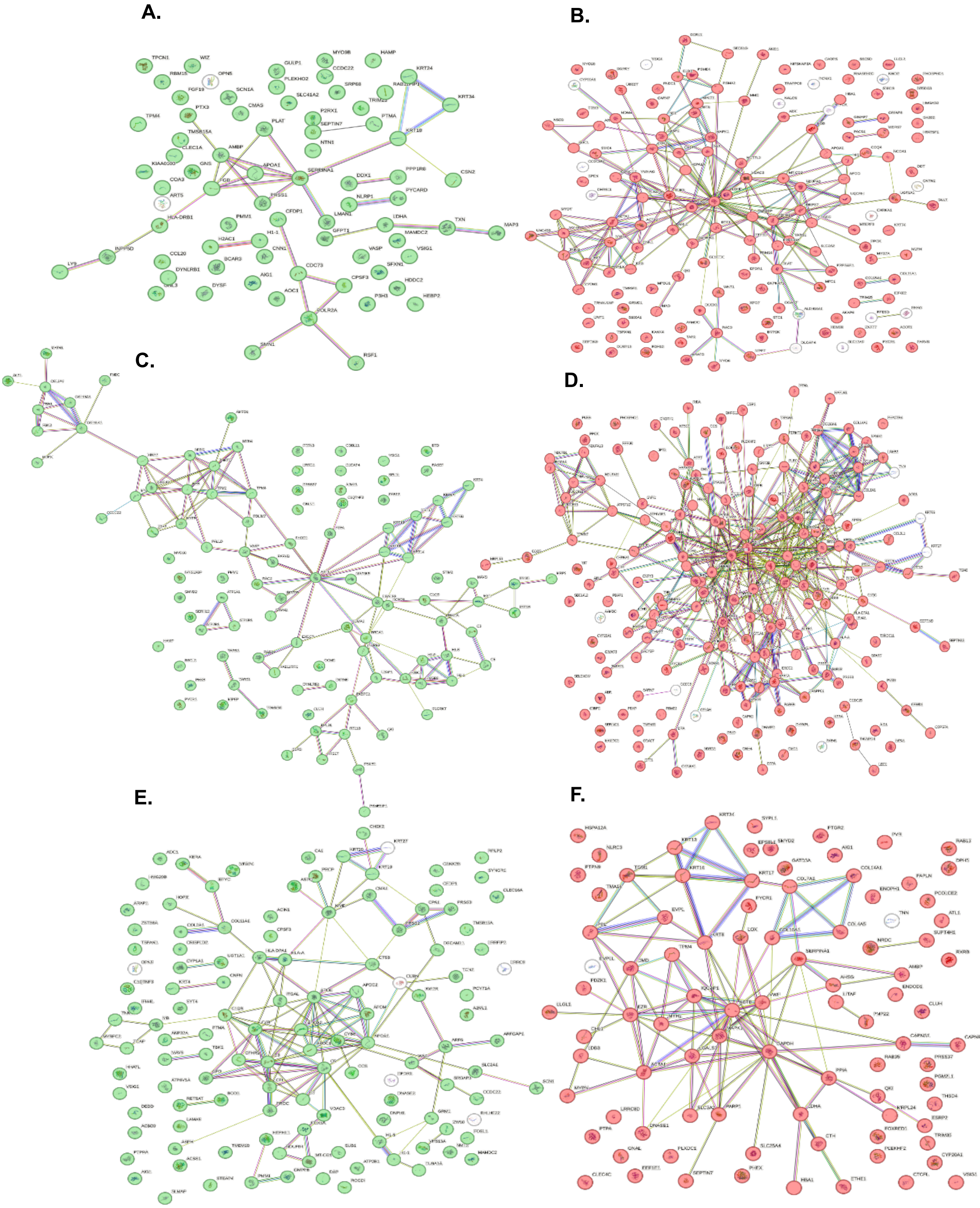
A.
B.
C.
D.
E.
F.
TPCN1
WIZ
RBM15
OPN5
FGF19
SCN1A
CMAS
PTX3
TPM4
TMSB15A
CLEC1A
AMBP
KIAA0100
GNS
COA3
APOA1
FGB
PRSS1
SERPINA1
PLAT
HLA-DRB1
PMM1
H2AC1
H1-1
CNN1
BCAR3
CCL20
DYSF
AIG1
AOC1
CFDP1
CDC73
POLR2A
SMN1
RSF1
CPSF3
P3H3
HDDC2
HEBP2
VASP
SFXN1
VSIG1
LDHA
MAMDC2
TXN
MAP3
GFPT1
LMAN1
NLRP1
PYCARD
DDX1
PPP1R8
CSN2
KRT18
KRT34
KRT24
PTMA
SEPTIN7
P2RX1
NTN1
SLC41A2
PLEKHO2
GULP1
MYO9B
CCDC22
SRP68
HAMP
TRIM22
INPP5D
LY9
HSPA12A
NLRC3
KRT31
SYPL1
SMYD2
PTGR2
PVR
RAB13
GATD3A
KRT13
KRT16
KRT17
COL14A1
DPH5
TGM1
COL7A1
PAPLN
PCOLCE2
ENOPH1
PYCR1
TNN
ATL1
EVPL
SUPT4H1
LOX
COL10A1
COL4A5
NRDC
RXRB
KRT8
AHSG
ENDOD1
CLUH
PDZK1
IQGAP1
VWF
EZR
MYH9
LDB3
GAPDH
CAPN8
RAB35
PRSS37
PGM2L1
ACTA1
PPIA
MYPN
SLC3A2
PARP1
DNASE1
QKI
MRPL24
THSD4
ESRP2
FOXRED1
PTPA
PLXDC1
SLC25A4
CTH
PLEKHF2
CYP20A1
CLEC4C
EEF1E1
PHEX
HBA1
ETHE1
CTCFL

Figure S4. Interactomes of proteins differentially expressed in gill tissue of *Gyrodactylus* infected responding guppies (*Poecilia reticulata*) on Day 13 post-infection. A. Interactome of the 167 proteins up-regulated in the responding guppies provided with the Apex® Branchia supplement over the control diet (number of nodes: 138, number of edges: 270, avg. local clustering coefficient: 0.412, PPI enrichment p=2.16e-06). **B.** Interactome of the 194 proteins down-regulated in the responding guppies provided with the Apex® Branchia supplement over the control diet (number of nodes: 159, number of edges: 181, avg. local clustering coefficient: 0.362, PPI enrichment p<1.0e-16). **C**. Interactome of the 167 proteins up-regulated in the responding guppies provided with the Apex® Branchia supplement over the control diet (number of nodes: 141, number of edges: 117, avg. local clustering coefficient: 0.331, PPI enrichment p-value: 0.0245). **D.** Interactome of the 363 proteins down-regulated in the responding guppies provided with the Apex® Branchia supplement over the control diet (number of nodes: 296, number of edges: 905, avg. local clustering coefficient: 0.375, PPI enrichment p<1.0e-16). **E.** Interactome of the 93 proteins up-in the resistant guppies provided with the Apex® Branchia supplement over the control diet (number of nodes: 74, number of edges: 39, avg. local clustering coefficient: 0.355, PPI enrichment p=0.003). **F.** Interactome of the 228 proteins down-regulated in the resistant guppies provided with the Apex® Branchia supplement over the control diet (number of nodes: 195, number of edges: 791, avg. local clustering coefficient: 0.506, PPI enrichment p<1.0e-16).

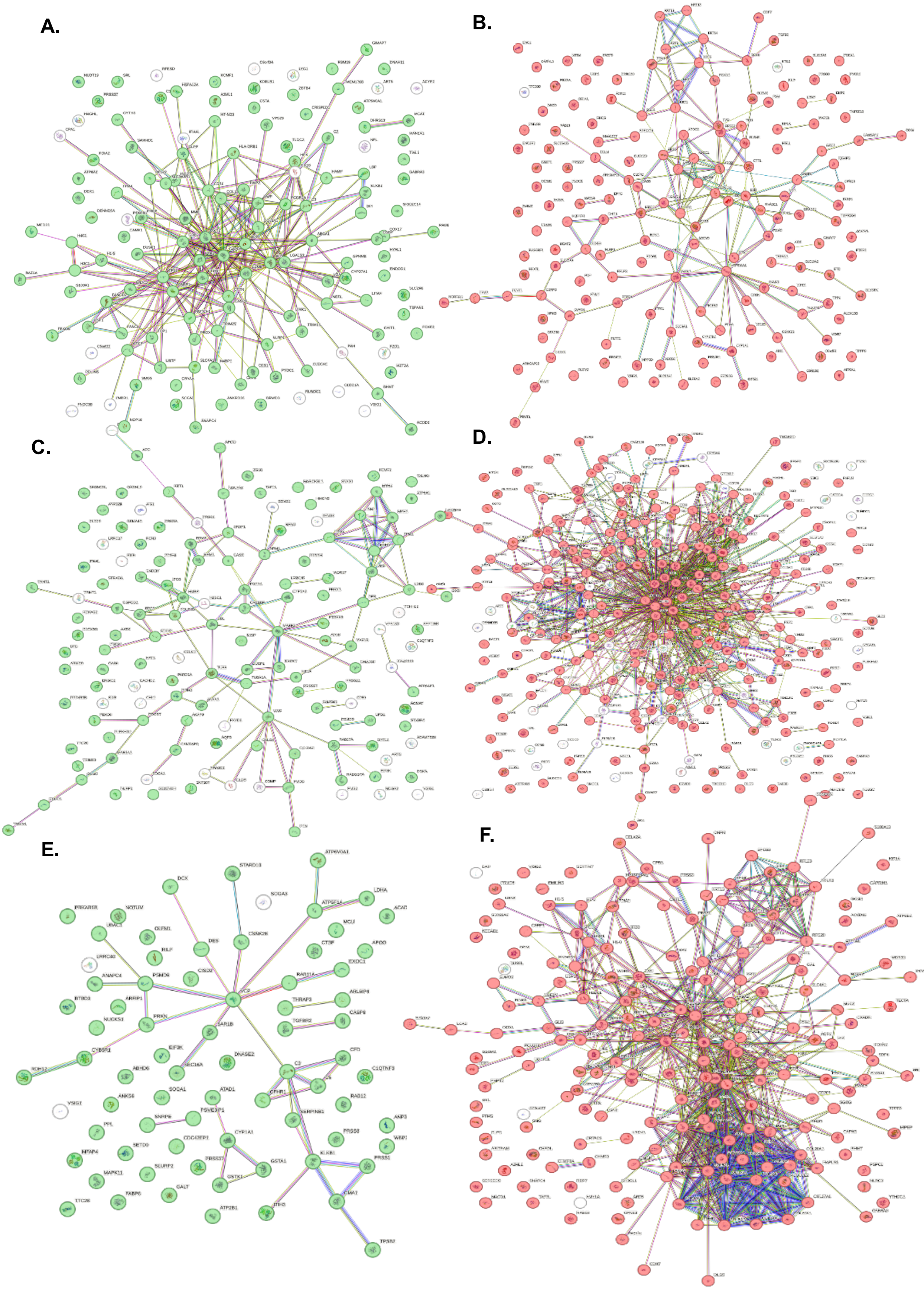
A.
B.
C.
D.
E.
F.

Figure S5. Interactomes of proteins differentially expressed in skin tissue of *Gyrodactylus* infected resistant guppies (*Poecilia reticulata*) on Day 17 post-infection. **A**. Interactome of the 131 proteins up-regulated in the responding guppies provided with the Apex® Branchia supplement over the control diet (number of nodes: 104, number of edges: 112, avg. local clustering coefficient: 0.388, PPI enrichment p=1.72e-08). **B.** Interactome of the 108 proteins down-regulated in the responding guppies provided with the Apex® Branchia supplement over the control diet (number of nodes: 98, number of edges: 113, avg. local clustering coefficient: 0.401, PPI enrichment p=0.0005). **C.** Interactome of the 111 proteins up-regulated in the resistant guppies provided with the Apex® Branchia supplement over the control diet (number of nodes: 95, number of edges: 111, avg. local clustering coefficient: 0.373, PPI enrichment p=0.002). **D.** Interactome of the 137 proteins down-regulated in the resistant guppies provided with the Apex® Branchia supplement over the control diet (number of nodes: 123, number of edges: 128, avg. local clustering coefficient: 0.367, PPI enrichment p=5.92e-12).

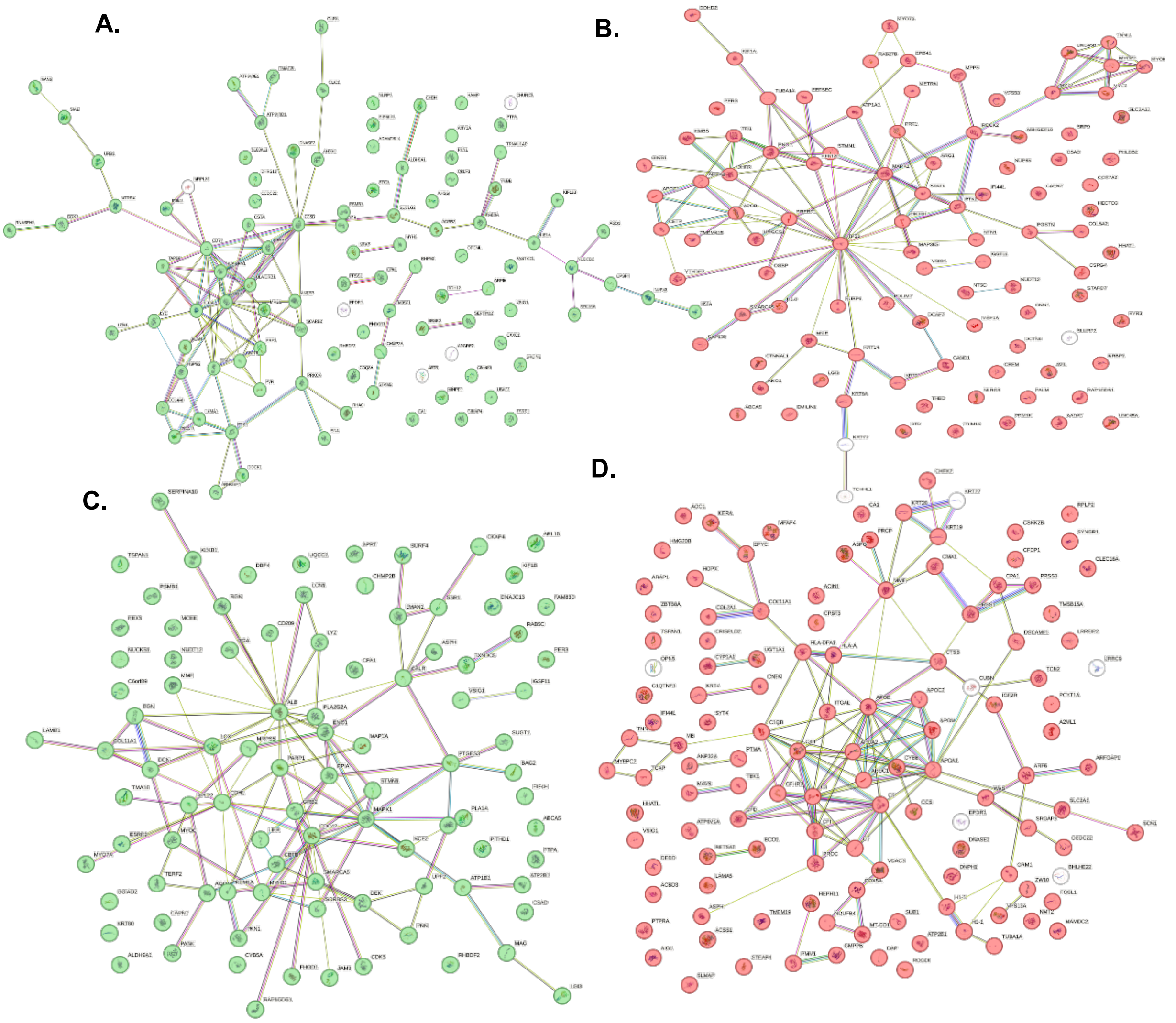

Figure S6. Interactomes of proteins differentially expressed in gill tissue of *Gyrodactylus* infected responding guppies (*Poecilia reticulata*) on Day 17 post-infection. **A**. Interactome of the 241 proteins up-regulated in the responding guppies provided with the Apex® Branchia supplement over the control diet (number of nodes: 197, number of edges: 253, avg. local clustering coefficient: 0.402, PPI enrichment p= 0.066). **B.** Interactome of the 208 proteins down-regulated in the responding guppies provided with the Apex® Branchia supplement over the control diet (number of nodes: 187, number of edges: 231, avg. local clustering coefficient: 0.38, PPI enrichment p=3.34e-06). **C**. Interactome of the 267 proteins up-regulated in the resistant guppies provided with the Apex® Branchia supplement over the control diet (number of nodes: 223, number of edges: 505, avg. local clustering coefficient: 0.393, PPI enrichment p=2.22e-16). **D.** Interactome of the 272 proteins down-regulated in the resistant guppies provided with the Apex® Branchia supplement over the control diet (number of nodes: 237, number of edges: 386, avg. local clustering coefficient: 0.384, PPI enrichment p=6.01e-07).

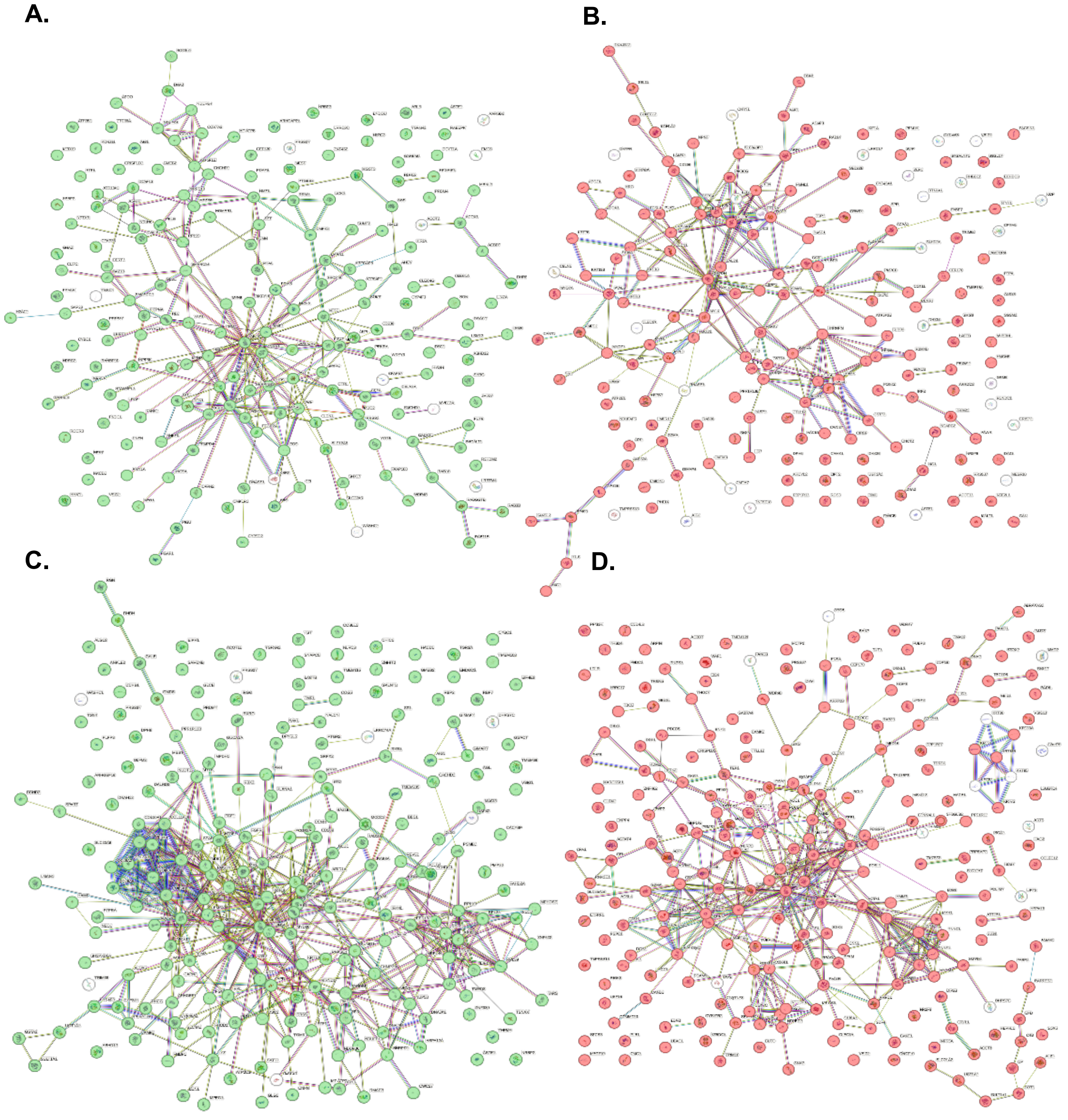

Supplement: Supplementary file 1 [file animals-16-01348-s001.zip › animals-4241264-supplementary.pdf]
